# Frequency of screening and SBT Technique Trial—North American Weaning Collaboration (FAST-NAWC): an update to the protocol and statistical analysis plan

**DOI:** 10.1186/s13063-023-07079-5

**Published:** 2023-10-02

**Authors:** Karen E. A. Burns, Myriam Lafrienier-Roula, Nicholas S. Hill, Deborah J. Cook, Andrew J. E. Seely, Bram Rochwerg, Michael Mayette, Frederick D’Aragon, John W. Devlin, Peter Dodek, Maged Tanios, Audrey Gouskos, Alexis F. Turgeon, Pierre Aslanian, Ying Tung Sia, Jeremy R. Beitler, Robert Hyzy, Gerard J. Criner, Elias Baedorf Kassis, Jennifer L. Y. Tsang, Maureen O. Meade, Janice M. Liebler, Jessica T. Y. Wong, Kevin E. Thorpe, For the Canadian Critical Care Trials Group

**Affiliations:** 1 Interdepartmental Division of Critical Care, University of Toronto, Toronto, Canada; 2 Division of Critical Care Medicine, St Michael’s Hospital, Toronto, Canada; 3 Li Ka Shing Knowledge Institute, St. Michael’s Hospital, 30 Bond Street, Office 4-045 Donnelly Wing, Toronto, ON M5B 1W8 Canada; 4 Applied Health Research Centre, St. Michael’s Hospital, Toronto, Canada; 5 Division of Pulmonary, Critical Care and Sleep Medicine, Tufts Medical Center, Boston, USA; 6 Department of Health Research Methods, Evidence and Impact, McMaster University, Hamilton, Canada; 7 Ottawa Hospital Research Institute, University of Ottawa, Ottawa, Canada; 8 Department of Medicine, McMaster University, Hamilton, Canada; 9 Centre Hospitalier Universitaire de Sherbrooke, Sherbrooke, Canada; 10 Centre de Recherche du Centre Hospitalier, Universitaire de Sherbrooke, Sherbrooke, Canada; 11 Bouve College of Health Professions, Northeastern University, Boston, USA; 12 Division of Pulmonary and Critical Care Medicine, Brigham and Women’s Hospital, Boston, MA USA; 13 Centre for Health Evaluation and Outcome Sciences, Vancouver, Canada; 14 University of British Columbia, Vancouver, Canada; 15 Pulmonary and Critical Care Medicine, Memorial Care, Longbeach Medical Center, Longbeach, CA USA; 16 Patient and Family Advisory Committee and Steering Committee Representative, FAST-NAWC Trial, Toronto, Canada; 17 Departments of Anesthesia and Critical Care, Hôpital Enfant-Jésus du CHU de Québec-Université Laval, Quebec City, Canada; 18 Service de Soins Intensifs, Département de Médecine, Centre Hospitalier de L’Universite de Montreal, Montreal, Canada; 19 Department of Critical Care Medicine, Centre Integre Universitaire de Sante et de Services Sociaux de la Mauricie-et-du-Centre-du-Quebec – Trois Rivieres, Montreal, Canada; 20 Center for Acute Respiratory Failure and Division of Pulmonary, Allergy, and Critical Care Medicine, Columbia University College of Physicians and Surgeons and New York-Presbyterian Hospital, New York, NY USA; 21 Division of Pulmonary and Critical Care, University of Michigan Health System, Ann Arbor, MI USA; 22 Division of Pulmonary and Critical Care Medicine, Temple University, Lewis Katz School of Medicine, Philadelphia, USA; 23 Departments of Medicine (Division of Critical Care) and Anesthesia, Beth Israel Deaconess Medical Center, Boston, USA; 24 Department of Medicine, Division of Critical Care, Niagara Health System – St. Catherines, St. Catherines, Canada; 25 Division of Critical Care, Hamilton Health Sciences Center, Hamilton, Canada; 26 Divisions of Pulmonary, Critical Care, and Sleep Medicine, Keck School of Medicine, University of Southern California, Los Angeles, USA; 27 Faculty of Medicine and Dentistry, University of Toronto, Toronto, Canada; 28 Institute for Health Policy, Management and Evaluation, University of Toronto, Toronto, Canada

**Keywords:** Weaning, Spontaneous breathing trial, Screening, Randomized controlled trial, Successful extubation

## Abstract

**Background:**

This update summarizes key changes made to the protocol for the Frequency of Screening and Spontaneous Breathing Trial (SBT) Technique Trial—North American Weaning Collaborative (FAST-NAWC) trial since the publication of the original protocol. This multicenter, factorial design randomized controlled trial with concealed allocation, will compare the effect of both screening frequency (once vs. at least twice daily) to identify candidates to undergo a SBT and SBT technique [pressure support + positive end-expiratory pressure vs. T-piece] on the time to successful extubation (primary outcome) in 760 critically ill adults who are invasively ventilated for at least 24 h in 20 North American intensive care units.

**Methods/design:**

Protocols for the pilot, factorial design trial and the full trial were previously published in J Clin Trials (https://doi.org/10.4172/2167-0870.1000284) and Trials (https://doi: 10.1186/s13063-019–3641-8). As planned, participants enrolled in the FAST pilot trial will be included in the report of the full FAST-NAWC trial. In response to the onset of the coronavirus disease of 2019 (COVID-19) pandemic when approximately two thirds of enrollment was complete, we revised the protocol and consent form to include critically ill invasively ventilated patients with COVID-19. We also refined the statistical analysis plan (SAP) to reflect inclusion and reporting of participants with and without COVID-19. This update summarizes the changes made and their rationale and provides a refined SAP for the FAST-NAWC trial. These changes have been finalized before completion of trial follow-up and the commencement of data analysis.

**Trial registration:**

Clinical Trials.gov NCT02399267.

**Supplementary Information:**

The online version contains supplementary material available at 10.1186/s13063-023-07079-5.

## Update

This is an update to the protocol for the Frequency of Screening and Spontaneous Breathing Trial (SBT) Technique Trial—North American Weaning Collaborative (FAST-NAWC) trial, which was originally published in *Trials* [1]. As originally planned, participants in the pilot factorial design FAST trial [2] will be reported on in the FAST-NAWC trial.

The FAST-NAWC trial is a multicenter, factorial design randomized controlled trial with concealed allocation, that will compare the effect of both screening frequency [once daily (OD) vs. at least twice daily (ALTD)] to identify candidates to undergo a spontaneous breathing trial (SBT) and SBT technique [pressure support (PS) + positive end-expiratory pressure (PEEP) vs. T-piece] on the time to successful extubation (primary outcome) in 760 critically ill adults who are invasively ventilated for at least 24 h in 20 North American intensive care units (ICUs). In the OD arm, respiratory therapists (RTs) will screen study patients between 06:00 and 08:00 h. In the ALTD arm, patients will be screened at least twice daily between 06:00 and 08:00 h and between 13:00 and 15:00 h with additional screens permitted at clinician’s discretion. When an SBT screen is passed, an SBT will be conducted using the assigned technique (PS + PEEP or T-piece). We will follow patients until successful extubation, death, ICU discharge, or until day 60 after randomization. We will contact patients or their surrogates 6 months after randomization to assess health-related quality of life (HRQoL) and functional status [1].

The primary objectives of the FAST-NAWC trial are to compare the effects of the alternative: screening frequencies (OD vs. ALTD) and the different SBT techniques (PS + PEEP vs. T-piece) on the time to successful extubation. This outcome is important to clinicians and citizens because it signals timely and safe liberation from the ventilator [3–5]. We defined successful extubation as the time when unsupported, spontaneous breathing began and was sustained for ≥ 48 h after extubation or disconnection in patients with tracheostomy [1]. The secondary objectives are to compare the effect of screening frequencies and SBT techniques on time to first passing an SBT, ICU mortality, hospital and 90-day mortality [6], total duration of mechanical ventilation, ICU and hospital length of stay, use of noninvasive ventilation after extubation, ventilator associated pneumonia, adverse events, self-extubation, tracheostomy, reintubation, prolonged ventilation at days 14 and 21, ICU readmission [7, 8], proportion receiving sedatives or analgesics or antipsychotics or screened positive for delirium at key time points, health related-quality of life (HRQoL) using the EuroQol EQ-5D, and a functional status assessment using the Functional Independence Measure (FIM) assessed at 6 months after randomization [9–12].

In response to the coronavirus disease of 2019 (COVID-19) pandemic when approximately two-thirds of enrollment was complete, we revised sections of the FAST-NAWC trial protocol [13, 14]. To reflect inclusion of critically ill patients who meet eligibility criteria and have COVID-19 infection, we modified specific sections of the protocol including the study population, conduct of SBTs, and the statistical analysis. In the text below, we highlight all protocol modifications made and the rationale for each revision. In parallel, based on the suggestion of the central Research Ethics Board (St. Michael’s Hospital, Toronto, Canada) and recognizing that COVID-19 status is a reportable illness, we modified the consent form to inform participants that trial data would be analyzed based on COVID-19 status. In this update, we also present an updated statistical analysis plan (SAP) for the FAST-NAWC trial given the addition of COVID-19 positive patients in the trial.

The COVID-19 pandemic occurred as the FAST-NAWC trial was approximately two thirds complete. At this time, considerable uncertainty existed in the critical care community regarding the ventilator course and outcomes of critically ill patients with COVID-19 who required invasive mechanical ventilation. Specifically, concern existed that invasively ventilated patients with COVID-19 would have worst clinical outcomes (e.g., longer duration of mechanical ventilation, higher reintubation rate, increased tracheostomy rate, and longer ICU stays) compared to invasively ventilated patients without COVID-19. Moreover, critically ill COVID-19 positive patients may be managed differently (e.g., increased use of prone positioning or neuromuscular blocker infusions) and clinicians may be more reluctant to conduct SBTs or extubate these patients due to concerns related to virus transmission and extubation failure. Given the uncertainty regarding the potential for COVID-19 to impact upon the primary and secondary trial outcomes, the Steering Committee of the FAST-NAWC trial, in consultation with the members of the Canadian Critical Care Trials Group (CCCTG), decided to continue to recruit and report upon the effect of the alternative interventions (screening frequency and SBT technique) in invasively ventilated patients without COVID-19. Concurrently, however, we also sought to understand the illness trajectory of invasively ventilated patients with COVID-19 and the impact of the trial interventions for patients with COVID-19. Therefore, we modified the protocol to include enrollment of up to 250 additional critically ill patients who tested positive for COVID-19 alongside the originally planned 760 (COVID-19 negative) patients. The ability to enroll up to 250 additional patients was permitted by reallocation of trial funds that would not be used for on-site monitoring visits due to pandemic and visitor restrictions at participating hospitals.

With regard to SBT conduct, once participants pass a screening assessment, they were expected to undergo an SBT according to the assigned treatment. We modified the trial protocol, to specify that COVID-19 negative patients would undergo an initial SBT according to treatment assignment using the 2 techniques that are most commonly utilized in practice internationally (PS + PEEP vs. T-piece) and being evaluated in the FAST-NAWC trial [1, 15–17]. We specified that COVID-19 positive patients would undergo SBTs with either [no support on the ventilator [(i.e., PS = 0 cm H_2_O + PEEP = 0 cm H_2_O or CPAP = 0 cm H_2_O) or PS + PEEP SBTs]. Regardless of the SBT technique utilized, SBT duration remained at 30–120 min in duration [14, 15] at the discretion of clinicians in all trial arms. The rationale for permitting COVID-19 trial participants randomized to T-piece SBTs to remain on the ventilator during SBTs was due to concerns infection control. Specifically, concerns existed regarding the potential for aerosolization of COVID-19 virus during conventionally performed T-piece trials in which the patient is disconnected from the ventilator. Consequently, trial participants who had COVID-19 infection would not be disconnected from the ventilator but rather would remain connected to the ventilator without aninspiratory or expiratory support during SBTs. (Additional file 1).

The COVID 19-related protocol revisions have implications for our original statistical analysis, wherein we stated that the primary, secondary, exploratory, and adjusted analyses would report on 760 COVID-19 negative patients, as originally intended. Given the potential for COVID-19 patients to have different ventilator trajectories and consequently clinical outcomes, we felt that we should analyze patients with and without COVID-19 separately. In this manner, we planned to report on weaning and clinical outcomes in COVID-19 positive and COVID-19 negative patients combined and separately. Specifically, we planned to report the primary and secondary outcomes in COVID-19 positive patients alone and with both cohorts (COVID-19 positive and negative) combined. The full revised trial protocol may be requested from the corresponding author via electronic mail. We also made updates and clarifications to the SAP in consultation with our trial statisticians (MF-R, KT). We present the detailed SAP for the FAST-NAWC trial in Additional file 2, which has not previously been published, alongside a completed SAP checklist in Additional file 3 in accordance with the Guidelines for the Content of Statistical Analysis Plans in Clinical Trials [18].

Clinicians and researchers do not know if invasively ventilated patients who have COVID-19 are different from invasively ventilated patients without COVID-19 infection and whether they will respond similarly to the different screening frequencies and SBT techniques being evaluated in the FAST-NAWC trial. The revised protocol may help to address that knowledge gap.

## Trial status

Protocol version/date: v6 dated 22 June 2020.

Recruitment FAST pilot trial start date: 15 June 2016.

Recruitment FAST pilot trial end date: 10 December 2016.

Recruitment FAST-NAWC trial start date: 22 January 2018.

Recruitment FAST-NAWC trial (COVID-19 negative) end date: 23 February 2022.

Recruitment FAST-NAWC trial (COVID-19 positive) end date: 23 August 2022.

Follow-up end date: 30 November 2022.

### Supplementary Information

**Additional file 1: Appendix 1.** FAST-NAWC Statistical Analysis Plan.

**Additional file 2: Appendix 2.** SAP checklist in accordance with the Guidelines for the Content of Statistical Analysis Plans in Clinical Trials.

**Additional file 3. **

## Data Availability

Not applicable.

## Abbreviations

FAST-NAWC: Frequency of Screening and Spontaneous Breathing Trial Technique Trial—North American Weaning Collaborative
SBT: Spontaneous breathing trial
PS: Pressure Support
PEEP: Positive end-expiratory pressure
ICU: Intensive care unit
CCCTG: Canadian Critical Care Trials Group
SAP: Statistical analysis plan
OD: Once daily
RT: Respiratory therapist
ALTD: At least twice daily
HRQoL: Health-related quality of life
FIM: Functional independence measure
USA: United States of America
CPAP: Continuous positive airway pressure
