# Supplementary material for: Frequency of screening and SBT Technique Trial—North American Weaning Collaboration (FAST-NAWC): an update to the protocol and statistical analysis plan
Source: Trials. 2023 Oct 2;24:626. doi: 10.1186/s13063-023-07079-5 (PMC10544476; doi:10.1186/s13063-023-07079-5)
Supplement: Supplementary file 1 — Additional file 1: Appendix 1. FAST-NAWC Statistical Analysis Plan. [file 13063_2023_7079_MOESM1_ESM.pdf]

## The Frequency of Screening and SBT Technique Trial: The FAST Trial

### *A North American Weaning Collaboration*

**1.0 What is the Challenge to be Addressed?**

Invasive mechanical ventilation is the archetype ICU life support technology. Most patients admitted to adult ICUs require invasive ventilation. The use of invasive mechanical ventilation in critically ill patients can be lifesaving. Although patients who receive life support technology interventions account for only 5-10% of acute care bed occupancy they consume 34% of hospital budgets [1]. These estimates are expected to increase in the near future amidst an aging middle sector of our population and patient and family expectations for enhanced longevity [2]. Health services research suggests that future demands may outstrip current Intensive Care Unit (ICU) capacity (personnel and life support equipment) [2,3].

Weaning is the process during which the work of breathing is transferred from the ventilator back to the patient. Approximately 40% of the time spent on invasive mechanical ventilation is consumed by weaning [4]. Although invasive ventilation is effective, it is associated with the development of numerous complications including respiratory muscle weakness, upper airway injury, ventilator associated pneumonia (VAP) [5] and sinusitis [6]. The incidence of VAP increases exponentially after day 5 of invasive ventilation and is associated with increased morbidity and a trend toward increased mortality [7]. *Limiting the duration of invasive ventilation has been identified as a key research priority in critical care [8].*

More than two decades of research support the use of specific strategies to limit patient's exposure to invasive ventilation including the (i) use of multidisciplinary screening protocols [to identify candidates for a spontaneous breathing trial (SBT)] [9-11], (ii) conduct of SBTs [12-14] to test patient's ability to breathe with limited or no support, and (iii) use of specific modes [reductions in Pressure Support (PS)] or techniques [once daily SBTs with PS ± positive end expiratory pressure (PEEP) or T-piece (no inspiratory or expiratory support)] [15-17] to decrease support in patients who fail an initial SBT. Compared to *usual care*, randomized trials and our meta-analysis suggest that protocolized weaning, led by allied health care providers [Respiratory Therapists (RTs) and nurses], reduced weaning time and ICU stay [12-14,18]. *Although a credible body of weaning evidence exists, it is insufficient to guide current practice.*

**1.1 The Weaning Paradox:** To improve outcomes and reduce complication rates, clinicians strive to shorten patients' exposure to invasive ventilation. However, premature or failed attempts at extubation necessitating reintubation are associated with morbidity (VAP) and an 8 to 10 fold increase in mortality [19]. *In their efforts to minimize patients' exposure to invasive ventilation, clinicians are challenged by a 'trade-off' between the risks associated with a premature failed attempt at extubation (too early) and the complications associated with protracted invasive ventilation (too late).*

**1.2 What are the Knowledge Gaps or Decisional Dilemmas in Weaning?**

**Dilemma #1) Clinicians Do Not Know How Often to Screen Patients to Identify SBT Candidates**

In a systematic review and meta-analysis of 17 trials (n=2,434), we demonstrated that the use of screening protocols to identify SBT candidates was associated with a 26% reduction in total duration of mechanical ventilation [n = 14 trials, 95% CI (13% - 37%), p = 0.0002], 70% reduction in weaning time [n = 8 trials, 95% (CI 27% - 88%), p = 0.009] and an 11% reduction in ICU stay [n = 9 trials, 95% CI (3% - 19%), p = 0.01][20]. Of the 17 included trials, *only one trial* (n=385) compared twice daily screening led by RTs and bedside nurses to *usual care* and noted a significantly shorter duration of ventilation and a trend toward less VAP in the twice daily screening group [11]. *These screening trials had several methodologic weaknesses including enrollment of heterogeneous populations, exclusion of patients receiving vasopressor and/or sedative infusions and with a hemoglobin concentration < 100 g/L, limiting screening to 'once daily' in the intervention arm and comparing protocolized screening to 'usual care' (control arm) requiring a physician order to conduct an SBT.*

**Dilemma #2) Clinicians Do Not Know the Best Way to Conduct an SBT**

After identification, clinicians conduct SBTs to determine if patients can breathe on low or no support. SBTs can be conducted using different techniques and for variable durations of time (typically 30 minutes to 120 minutes). In the absence of a clinical practice guideline, *the Task Force on Weaning*, a

consensus statement, recommends that (i) weaning should be considered as early as possible, (ii) an SBT is *the major diagnostic* test to determine if patients can be successfully extubated, and (iii) an initial SBT should be 30 minutes in duration and be conducted with T-piece or low levels of PS (5–8 cm $H_2O$ in adults) ± 5 cm $H_2O$ PEEP [21]. *The Task Force highlights the need for additional research to be conducted evaluating specific aspects of weaning protocols that improve outcomes. The FAST Trial directly addresses this call for research by evaluating screening frequency and SBT technique concurrently in a single trial.*

Among patients deemed 'ready to wean' and in whom vasopressors and sedatives were discontinued, 2 large trials that compared 2 SBT techniques [T-piece (no support) vs. PS 7 cm $H_2O$ (minimal support to overcome resistance of the breathing tube)] and 2 different SBT durations (30 vs. 120 minutes) found no significant differences in the proportion of patients successfully extubated [12,13]. These trials suggest that clinicians can conduct SBTs using either PS or a T-piece and that a 30 minute SBT is sufficient for most patients. *Several factors limit the inferences that can be made from these trials to current practice. First, both trials predated daily screening and patients in these trials were deemed 'ready for weaning' by physicians. Second, these trials were conducted in Europe and South America where weaning is directed solely by physicians rather than in collaboration with RTs as is standard practice in North American ICUs. Third, neither trial was powered to identify differences in clinically important clinical outcomes.*

Two systematic reviews, including a Cochrane review and our meta-analysis have compared T-piece and PS SBTs with discordant results [22,23]. In 4 trials of moderate quality, Ladiera and coworkers found that PS was significantly superior to T-piece for successful SBTs [Risk Ratio (RR) 1.09, 95% CI 1.02 to 1.17] [22]. Conversely, in 9 trials (n=1,901) we found that patients undergoing PS (vs. T-piece) SBTs were *as likely* to pass an initial SBT [Risk Ratio (RR) 1.00, 95% confidence interval (CI) 0.89-1.11; $I^2$ = 77%] (moderate quality evidence) but were *significantly more likely to* be successfully extubated [RR 1.06, 95% CI 1.02-1.10; 11 trials, n=1,904; $I^2$=0%, NNT =19.7] (moderate quality evidence) [23]. Exclusion of one trial with inconsistent results for SBT and extubation outcomes supported that PS (vs. T-piece) SBTs improved SBT success [RR 1.06, 95% CI 1.01-1.12; $I^2$ = 0%]. Subgroup analysis showed higher SBT success in non-perioperative vs. perioperative trials although between-group differences were not significant [23]. No trial comparing these SBT techniques was conducted in North America. *Evidence from RCTs and meta-analyses have not identified the best way to test patient's ability to be breathe spontaneously before extubation – one of the most important decisions made for critically ill patients. Current best evidence predates once daily screening, may not be generalizable to North American ICUs, and is poorly aligned with patients' and families' desire to be 'successfully extubated' in a timely, effective, and safe manner [24].*

**Dilemma #3) Clinicians Do Not Know How to Discontinue Support in Patients Who Fail an Initial SBT**

Few trials have compared alternative strategies for discontinuing support for patients who require repeated SBTs. In 1994, Brochard and coworkers in France randomized patients to reductions of PS (n=31), titration of synchronized intermittent mandatory ventilation (SIMV) rate (n=43), or once daily SBTs with T-piece (n=35) and found that PS was superior to the *combined analysis* of SIMV and once daily SBTs (using T-piece) with regard to weaning duration, the probability of remaining on mechanical ventilation, and ICU stay, however, differences among the three arms were not significant [15]. Subsequently, Esteban and colleagues in Spain compared 4 different weaning strategies [reductions of PS (n=37), titration of intermittent mandatory ventilation (IMV) rate (n=29), once daily SBTs with T-piece (n=31), and multiple daily SBTs with T-piece (n=33)] [16]. Comparing differences *among strategies*, the authors found that patients who had once daily SBTs were 2.83 [95% CI (1.36 - 5.89); $p < 0.006$] and 2.05 [95% CI (1.04 - 4.04); $p < 0.04$] times more likely to be successfully weaned compared to patients who had reductions in IMV and PS, respectively. They found no difference in successful weaning between SBTs conducted once daily vs. multiple times daily RR 1.24 [95% CI (0.64 - 2.41); p=0.54]. Both trials permitted use of small amounts of PEEP [$\leq$ 4 cm $H_2O$ [15] and $\leq$ 5 cm $H_2O$ [16], respectively] to offset intrinsic PEEP and facilitate ventilator triggering. *The Esteban study changed weaning from a process of gradually reducing support on conventional weaning modes to a process of 'discovering' the earliest time when patients can breathe spontaneously using SBTs [16]. Current use of serial SBTs (once daily vs. multiple times daily) as a weaning strategy for patients who fail an initial SBT and are at highest risk of adverse outcomes is based on 2 RCTs involving 99 patients.*

### 1.3 Preparatory Work for the FAST Trial

In preparation for the FAST Trial, we conducted a national survey, an international survey of weaning practices and 2 pilot trials (n=156) comparing ‘once daily’ vs. ‘at least twice daily’ screening.

1.3.1 *In a National Weaning Survey of Canadian teaching hospitals*, *we found that 95.6% of Critical Care site chiefs and head RTs stated that they screened once daily to identify SBT candidates and only 32% of respondents screened twice daily [25]. We also identified that clinicians in Canada commonly use 3 techniques (CPAP, PS, and T-piece) to conduct SBTs [25].*

*1.3.2 In an International Weaning Survey* involving 1,142 intensivists in Canada, the United States of America (USA), United Kingdom(UK), India, Europe and Australia/New Zealand, we found that most respondents frequently, usually or always conduct *once daily screening* (range 70%-96% across regions) to identify SBT candidates**,** especially in the US (96.0%) and Canada (92.7%) [26]. Twice daily screening to identify SBT candidates was used less often (range 12.2% - 33.9% across regions) and *more than twice daily screening* was rarely used (range 1.6% - 18.2% across regions). In both the national and international weaning surveys, we found that PS alone (gradual titration) or PS with SBTs (titration plus SBTs) were the most frequently used weaning techniques. Internationally, we identified that clinicians predominantly use 2 techniques (PS and T-piece) to conduct SBTs [26]. *Our findings suggest that ‘once daily’ screening to identify SBT candidates is the current standard of care in weaning with 24 hours typically elapsing between screening periods and that PS and T-piece are most commonly used SBT techniques.*

Regional differences in practice may be explained, in part, by the different roles played by various health care providers involved in weaning across geographic regions. For example, in Canada and the USA, RTs screen patients to identify SBT candidates and conduct SBTs. Conversely, physicians in India or physicians and nurses in the UK, Europe, and Australia/NewZealand perform screening and actually conduct SBTs. Similarly, whereas RTs and physicians work collaboratively in North American ICUs to titrate ventilator settings, physicians and nurses titrate ventilator settings in Australia/New Zealand and the UK. Across regions, physicians preserved decisional authority regarding extubation and tracheostomy [26]. *Our survey highlights that weaning evidence may not be readily generalizable from one region to another as different health care providers are involved in implementing evidence into practice***.**

*1.3.3 Pilot Screening Frequency Trials in Non-Elderly and Elderly Patients*

Due to concerns that fewer elderly (vs. younger) critically ill patients may be enrolled in weaning trials because of more frequent comorbidities, treatment limitations, and increased potential for adverse events, we secured 2 grants to conduct 2 pilot RCTs to evaluate screening frequency (once vs. at least twice daily screening) in 100 elderly patients ≥ 65 years in 11 ICUs (SENIOR Trial; ClinicalTrials.gov NCT02243449) and 50 non-elderly patients < 65 years in 10 ICUs (RELEASE Trial; NCT02001220) [27] in parallel. We prioritized evaluation of screening frequency in our pilot trials recognizing that a ‘change in screening culture’ warranted a rigorous, multicenter feasibility assessment. We permitted centres to use their preferred SBT technique in both pilot trials because the SBT techniques used at participating centres were among the commonly used SBTs techniques nationally and internationally.

In the parallel pilot trials, we demonstrated our ability to recruit (1.32 and 1.26 patients per ICU in the RELEASE and SENIOR trials respectively, or 2.58 patients per ICU per month combined) and consent (91.1% and 90.7%, in RELEASE and SENIOR, respectively) the desired population*.* Our high consent rates likely reflect 2 factors: the low-risk interventions being evaluated in our trials and approval from all Canadian research ethics boards (REBs) to use a hybrid consent model. In this model, consent from patients and substitute decision makers (SDMs) was prioritized, however, patients could be enrolled using deferred consent [28] if patients were not capable of providing primary consent and SDMs were not available given the time sensitive nature of enrolment (i.e., before patients were placed on SBT equivalent settings) [27]. *We noted acceptable protocol violation rates (< 20%), few related and unrelated adverse events (< 5%), no serious adverse events, and similar effects on clinical outcomes. We refined our trial exclusion criteria after demonstrating comparable rates and reasons for trial exclusion overall with slightly more SENIOR trial participants excluded for age-related illnesses [cardiac arrest (13.2 %) and myocardial infarction (4.7%)]. Collectively, the findings of the parallel pilot RCTs support trial feasibility and conduct of a single large weaning trial without age restriction* (see Preliminary Results Table below).

| Outcome | Release Trial Once Daily (n=23) | Release Trial At least Twice (n=31) | Senior Trial Once Daily (n=54) | Senior Trial At least Twice (n=49) |
|---|---|---|---|---|
| **ICU length of stay (median)** | **10 days** | **9 days** | **10 days** | **9 days** |
| **Hospital length of stay (median)** | **25 days** | **28 days** | **29 days** | **18 days** |
| **Time to successful extubation (median)** | **5.0 days** | **3.76 days** | **6.83 days** | **3.10 days** |
| **ICU mortality** | **6 (26.1%)** | **4 (12.9%)** | **13 (24.1%)** | **11 (22.4%)** |
| **Hospital mortality** | **0** | **3 (9.7%)** | **4 (7.4%)** | **5 (10.2%)** |
| **Use of Noninvasive ventilation** | **3 (13.0%)** | **7 (22.6%)** | **5 (9.3%)** | **8 (16.3%)** |
| **Tracheostomy** | **2 (8.7%)** | **2 (6.5%)** | **3 (5.6%)** | **5 (10.2%)** |

1.3.4 Systematic Reviews and Meta-Analyses

In a Cochrane review that compared protocolized screening to usual care, we found beneficial effects to protocolized screening (vs. usual care) on duration of ventilation, weaning time and ICU stay [20]. In a large systematic review that compared alternative SBT techniques, we found that compared to T-piece, patients undergoing PS SBTs were 6% (NNT =19.7) more likely to be successfully extubated [23]

1.3.5 Interviewer-Administered Questionnaire of Outcomes Important to Citizens in Weaning Trials

To prioritize outcomes important to citizens, we conducted an interviewer-administered questionnaire of 322 visitors of critically ill patients in 2 family waiting rooms (serving 3 ICUs). Respondents rated the importance of general and ventilation-related outcomes in 2 hypothetical scenarios [before a first SBT, and after a failed SBT] and selected a preferred SBT technique in both scenarios [24]. For visitors, the most important ventilation-related outcomes were being ventilator-free (8.95), avoiding reintubation (8.905), and passing a spontaneous breathing trial (8.903). Passing a SBT assumed greater importance after an initial failed attempt. General outcomes of importance were ICU and hospital survival (9.72,9.70), avoiding complications (9.45), quality of life (9.394), patient comfort (9.393) and return to previous living arrangements (9.31)[24]. *These findings informed our selection of primary and secondary outcomes and highlighted the need for longitudinal patient follow-up in the FAST Trial.*

### 1.4 Why Is a Weaning Trial Needed? Summary of Justification

*1.* A large evidence gap exists regarding the best strategy (screening frequency, SBT technique) to use to liberate our sickest patients from ventilators and ensure timely and successful extubation.
2. This evidence gap results in significant practice variation and poses several dilemmas for clinicians.
3. Weaning is among the *top 5%* of research questions identified by relatives and ICU clinicians. Preventing lung damage during invasive ventilation was identified as the *number one* research priority for patients, families, and clinicians [29,30].
4. Although most critically ill patients undergo weaning, 'best evidence' is *not relevant to current practice.*
5. Once daily screening is poorly aligned with the 24/7 continuous care model provided in ICUs.
6. Uncertainty exists regarding the best 'test' (SBT technique) to use to evaluate patients' ability to breathe spontaneously and maximize their chance of being successfully extubated
7. Around the clock presence of RTs in North American ICUs presents a unique opportunity to identify the best weaning strategy (both screening frequency and SBT technique) to liberate patients from ventilators.
8. The interventions evaluated are simple, low-risk, and directly applicable to North American ICUs.

### 1.5 What are the Overall Goals of the FAST Trial and potential contributions?

*In the FAST Trial*, we will conduct a multicenter, factorial design RCT involving 760 critically ill adults in 20 ICUs in Canada and the USA comparing the effect of 2 different screening frequencies (once daily vs. at least twice daily) and 2 different SBT techniques [T-piece vs. PS + PEEP] on outcomes that are important to stakeholders (patients, family members, and clinicians) involved in weaning [24]**.**

**Goal 1: To identify the best strategy for clinicians to use to liberate critically ill adults from ventilators to improve the health outcomes of critically ill adults.**

**Goal 2: To inform clinical decision-making and enhance care delivery.**

**Goal 3: To build a structural framework for future trials through novel collaborations involving**:

i) Canadian and American patients (ICU survivors) and family representatives, RTs, and trialists

ii) Research Consortia [Canadian Critical Care Trials Group (CCCTG) and the NIH Prevention and Early Treatment of Acute Lung Injury (PETAL) network].
iii) North American respiratory care societies [Canadian Society for Respiratory Therapy (CSRT), American Association of Respiratory Care (AARC)]; critical care societies [Canadian Critical Care Society (CCCS), American Thoracic Society (ATS), Society of Critical Care Medicine (SCCM), and patient & family advocacy groups [Patient Advisory Roundtable (ATS) and the Patient & Family Partnership Committee (CCCTG)].

**1.6 How is the FAST Trial Novel?**

The FAST Trial is novel in several ways. First, the FAST Trial will be the largest weaning trial ever conducted in North America. Second, it will serve as a demonstration project on how to actively engage patients and families in all aspects of critical care trial design and implementation and will advance patient and family engagement research methodology. Third, the FAST Trial has been designed and will be implemented with novel collaborations between Canadian and American research networks, respiratory care and critical care societies, advocacy groups, patients (ICU survivors) and family members.

A highly innovative component of the FAST trial is the integration of ICU survivors and family members into a Patient and Family Advisory Committee (PFAC). Our PFAC members have direct knowledge and experience with mechanical ventilation. *Over the past 12 months, all PFAC members have participated in discussions related to the FAST trial design and primary outcome selection.* One *family member (MF) reviewed part of the protocol.* During trial implementation, PFAC members will (i) directly advise the Steering Committee on study design and implementation issues that are important to them, (ii) be represented on the trial Steering Committee and Data Safety and Monitoring Committee (DSMC), (iii) aid in preparing study materials (e.g., consent forms, brochures regarding weaning and trial participation) that are easy for family members to read and understand, (iv) aid in selecting a metric to assess functional status at 6 month follow-up assessments, (v) participate in Q2 monthly teleconferences during trial implementation, (vi) contribute to developing a moderated on-line space that will serve as a repository for patient and family narratives, (vii) coauthor publications (posters, manuscripts), and (viii) participate in dissemination activities. PFAC members will be paid for their time and invited to attend either an international scientific or research consortium meeting to enhance their understanding of clinical research.

**1.7 What are the Anticipated Results?**

a) The FAST Trial will identify the best strategy (screening frequency, SBT technique) for clinicians to use to liberate critically ill adults from ventilators, improve health care delivery and health outcomes.
b) The FAST Trial will introduce a new paradigm of patient and family engagement into critical care research methodology.
c) The FAST Trial will provide a structural framework for future mechanical ventilation collaborations.
The FAST Trial holds promise as a clinical trial that could transform weaning practices in ICUs.

**2.0 Research Approach and Methods**

**2.1a) Primary Objectives:** The primary objectives of the FAST trial are to demonstrate the effect of:
(i). The alternative screening strategies (‘once’ vs. ‘at least twice’ daily) on time to successful extubation.
(ii). The alternative SBT techniques (T-piece vs. PS + PEEP) on time to successful extubation.

**2.1b) Secondary Objectives*:*** In secondary objectives, we will obtain estimates of the impact of the alternative screening and SBT techniques on clinically important outcomes including time to first passing an SBT, ICU mortality, hospital and 90 day mortality [31], total duration of mechanical ventilation, ICU and hospital length of stay, use of noninvasive ventilation (NIV) after extubation, VAP, adverse events, self-extubation, tracheostomy, reintubation, prolonged ventilation at day14 and 21, ICU readmission [32,33], proportion receiving sedation/ analgesia/ antipsychotics or screened positive for delirium at key time points, health related quality of life (HRQoL)(EuroQuol EQ-5D) and a functional status assessment [the Functional Independence Measure (FIM)] assessed 6 months after randomization [34-37].

**2.2 'A Priori' Hypotheses:** *A priori,* we expect that patients who are screened more frequently will have a shorter time to successful extubation regardless of the SBT technique used. We do not expect SBT technique alone to influence the time to successful extubation or successful extubation rate.

**2.3 Study Population:** We will include 760 COVID-19 negative and up to 250 COVID-19 positive critically ill adults [$\geq$ 18 years of age (USA) or $\geq$ 16 years of age (Canada) or admitted to an adult ICU] who are receiving invasive ventilation for at least 24 hours in 20 North American ICUs. Based on our pilot trials, we expect to recruit, on average, 1 out of every 5 patients screened by research personnel (1 in 5 for RELEASE; 1 in 5.3 for SENIOR) or 2.58 patients/ICU per month over a 31 month recruitment period. To be conservative, we expect to recruit 2 patients/ICU per month.

**2.4 Inclusion Criteria**
(1) Receiving invasive mechanical ventilation for $\geq$ 24 hours.
(2) Capable of initiating *spontaneous* breaths or *triggering* the ventilator to give a breath on ventilator modes commonly used in the ICU.
(3) Fractional concentration of inspired oxygen ($FiO_2$) $\leq$ 70%.
(4) Positive End-Expiratory pressure (PEEP) $\leq$ 12 cm $H_2O$.

**2.5 Exclusion Criteria**
(1) Brain death or expected brain death.
(2) Evidence of myocardial ischemia in the 24 hour period before enrollment.
(3) Received continuous invasive mechanical ventilation for $\geq$ 2 weeks.
(4) Tracheostomy in situ at the time of screening.
(5) Receiving a sedative infusion(s) for seizures or alcohol withdrawal.
(6) Require escalating doses of sedative agents.
(7) Receiving neuromuscular blockers or who have known quadriplegia, paraplegia or 4 limb weakness or paralysis preventing active mobilization.
(8) Moribund (e.g., at imminent risk for death) or who have limitations of treatment.
(9) Profound neurologic deficits (e.g., post cardiac or respiratory arrest, large intracranial stroke or bleed) or Glasgow Coma Scale (GCS) $\leq$ 6.
(10) Use of ventilator modes that automate SBT conduct.
(11) Currently enrolled in a confounding study that includes a weaning protocol.
(12) Previous enrollment in this trial.
(13) Previous SBT or are already on T-piece, or CPAP alone (without PS), or PS $\leq$ 8 cm $H_2O$ regardless of PEEP, or other 'SBT equivalent' settings immediately before randomization.
(14) Previous extubation [planned, unplanned (e.g. self, accidental)] during the same ICU admission.

**2.6 Randomization and Consent**

Research personnel (research coordinators and/or RTs) will identify, consent (where applicable) and enroll eligible patients from Monday to Friday during regular hours using a central randomization system, stratified by ICU with variable undisclosed block sizes. Patients will be randomized to a screening frequency (once vs. at least twice daily) AND an SBT technique (PS + PEEP vs. T-piece). The day of randomization will be considered study day one.

Given the minimal risk associated with the interventions being evaluated (screening frequency and SBT conduct) and the need to enroll patients as soon as possible after they initiate spontaneous breaths or trigger breaths, we will request ethics approval to use a hybrid consent model that prioritizes patient (with decision-making capacity) or SDM consent (when available) and permits deferred consent in their absence.

**2.7 Study Interventions**

In the 'once daily screening arm', RTs will screen invasively ventilated patients between approximately 06:00 - 08:00 hours daily. In the 'at least twice daily' screening arm, patients will be screened at a minimum between approximately 06:00 - 08:00 hours and between 13:00 - 15:00 hours daily. If a

screening period is missed (inadvertently or due to an investigation or intervention (operation/procedure) necessitating absence from the ICU), it may be conducted later on the same day and ideally within 6 hours of the scheduled screening period. Additional screening trials in the 'at least twice daily' screening arm will be permitted at the discretion of clinicians caring for the patient. To standardize procedures, if a patient is randomized *before* 10:00 am, both screening strategies can be initiated on the day of randomization. For patients randomized *after* 10:00 am, only one screening assessment will be required on the first study day.

**2.7.1 Screening for SBT Readiness**

To pass a screening assessment and undergo an SBT, all of the following criteria must be met:

1. The patient must be capable of initiating spontaneous breaths on PS or PAV or triggering breaths on volume or pressure AC, volume or pressure SIMV ± PS, PRVC, VS or APRV,
2. The ratio of partial pressure of oxygen to $FiO_2$ ($PaO_2/FiO_2$) $\geq$ 200 mm Hg,
3. RR $\leq$ 35 breaths/min,
4. PEEP $\leq$ 10 cm $H_2O$,
5. HR $\leq$ 140 beats/min,
6. The ratio of respiratory frequency to tidal volume ($f/V_T$) < 105 breaths/min/L during a 2 minute assessment on CPAP of 0 cm $H_2O$ (alternatively PS= 0 cm $H_2O$ /PEEP = 0 cm $H_2O$).

In selecting screening criteria, we avoided arbitrary criteria (no sedation, no vasopressors, hemoglobin above 100 g/L, and subjective assessments of level of consciousness) that may delay progression to an SBT. We included an objective measurement, the rapid shallow breathing index ($f/V_T$), conducted on standardized settings [CPAP of 0 cm $H_2O$ (alternatively PS= 0 cm $H_2O$ /PEEP = 0 cm $H_2O$)] to determine progression to an SBT.

**2.7.2 Conduct of Spontaneous Breathing Trials**

After passing a screening assessment, COVID-19 negative patients will undergo an initial SBT according to treatment assignment (T-piece vs. PS + PEEP). COVID-19 positive patients will undergo an initial SBT with either [no support on the ventilator (i.e., PS=0 cm $H_2O$ + PEEP=0 cm $H_2O$ or CPAP = 0 cm $H_2O$ vs. PS + PEEP]. All SBTs will be 30-120 minutes in duration [12,21,27]. SBTs will be conducted on T-piece (off ventilator with no CPAP/PEEP) or with PS >0 and $\leq$ 8 cm $H_2O$ with PEEP> 0 and $\leq$ 5 cm $H_2O$. Between SBT trials, patients will be returned to the mode of ventilation used before the SBT, unless criteria are met to remain on/return to a mode of support that assumes no spontaneous or triggered breaths. We will use standardized criteria to determine SBT failure in both arms [38].

SBT failure/Termination criteria is defined by the presence of any ONE of:

a) a respiratory rate (RR) > 35 breaths/min with signs of respiratory distress or an increase in RR ≥ 20% from baseline with signs of respiratory distress
b) oxygen saturation of arterial blood ($SaO_2$) or pulse oximetry < 90%;
c) heart rate > 140 beats/min with signs of respiratory distress or an increase in HR ≥20% from baseline with signs of respiratory distress;
d) systolic blood pressure ≥ 180 or ≤ 90 mm Hg;
e) the presence of somnolence, agitation, diaphoresis, or anxiety;
f) requirement for the addition of or an increase in vasopressor or inotropic agent support; and
g) chest pain or other limiting pain precluding further continuation.

After an unsuccessful SBT, patients will be returned to the ventilator settings used prior to the SBT. Ventilator settings will be adjusted to restore respiratory comfort, recognizing that some patients may meet criteria to go on a supported mode of ventilation without spontaneous or triggered breaths

**2.7.3 Criteria to Suspend the Protocol and Return to a Controlled/Supported Mode of Ventilation**

Patients should remain on a mode that permits spontaneous or triggered breaths between SBTs and at night. In all groups, patients will be permitted to *return to/remain on a supported mode of ventilation without spontaneous or triggered breaths* when any of the following criteria are met:

1. Surgery or invasive procedures requiring sedation and/or analgesia,
2. Respiratory distress as defined by:

   a) sustained hypoxemia [pulse oximetry oxygen saturation ( $SpO_2$) < 90%] with an $FiO_2$ > 60% and PEEP > 10 cm $H_2O$ or hypercapnia with pH < 7.30 OR clinical respiratory distress,

b) Repeated episodes ($\geq$ 3 episodes within 1 hr) wherein an inspiratory pressure (drive pressure + PEEP on pressure modes or plateau pressure on volume modes) of 35 cm $H_2O$ or more is attained (despite suctioning, bronchodilation etc),

3. Hemodynamic instability despite fluid boluses and requirement for high dose vasopressors: norepinephrine > 15 µg/min (0.2 µg/kg/min) or equivalent,
4. Suspected myocardial ischemia based on EKG and/or elevated Troponin I,
5. Neurologic deterioration with need to control $PaCO_2$ (e.g., raised intracranial pressure) or central hypoventilation.
6. RR < 10 breaths/min related to need for increased sedation,
7. PEEP $\geq$ 13 cm $H_2O$,
8. $FiO_2 \geq$ 71%.

Patients meeting any of these criteria will be reassessed daily to identify the earliest time when they meet initial inclusion criteria and the screening and SBT protocols can be resumed as per initial treatment assignment. If patients can breathe spontaneously on PS or PAV, or trigger spontaneous breaths on volume or pressure AC, volume or pressure SIMV ± PS, PRVC, VS, or APRV before 10:00 am, the allocated screening protocol can resume. However if, patients can not breathe spontaneously (PS or PAV) or trigger breaths (on volume or pressure AC, volume or pressure SIMV ± PS, PRVC, VS, or APRV) until after 10:00 am, screening will be conducted only once daily in both treatment arms on the day of the return and will resume as per treatment allocation thereafter. Practices checklist assessments will not be mandatory while patients remain on a fully controlled mode.

**2.7.4 Extubation:** Patients who pass an SBT will be assessed for extubation using standardized criteria. Extubation should be performed as soon as possible after passing an SBT. To be extubated the following criteria [26,38] should be met

1) $SpO_2 \geq$ 90% or at baseline level in chronically hypoxemic patients on an $FiO_2 \leq$ 40% and PEEP $\leq$ 5 cm $H_2O$,
2) a cough of sufficient strength to clear secretions and must not require suctioning more than every 2 hours,
3) patients should be hemodynamically stable [off vasopressors or on minimal levophed i.e., $\leq$7 µg/min (0.1 µg/kg/min or equivalent),
4) a level of consciousness sufficient to ensure airway protection and
5) a cuff leak is present. All of the above criteria (except #4 and 5) will also apply to patients who undergo trach mask trials and are disconnected.

**2.7.5 Other Important Considerations (Noninvasive Ventilation, Reintubation and Tracheostomy)**

**(i) Ventilator Titration:** Approaches to titration of ventilator settings, PEEP and $FiO_2$ in both arms are detailed in Appendix 1. While on study protocol, RTs will assess and titrate ventilator settings, $FiO_2$, and PEEP levels at least every 4 to 6 hours.

**(ii) Use of NIV after extubation:** We developed standardized criteria to guide the use of NIV after extubation. For NIV to be used after extubation, patients must have successfully completed an SBT and been extubated. In the FAST Trial, patients cannot be extubated directly to NIV to facilitate weaning as this technique obviates the need for an SBT. While our meta-analysis supports extubating patients [especially those with chronic obstructive pulmonary disease (COPD)] to NIV, our survey found that few clinicians have adopted this strategy in practice [25,39]. See Appendix 1.

**(iii) Criteria for reintubation:** All patients requiring reintubation after successful extubation will be ventilated according to usual practice and at the discretion of the clinical team. If patients are reintubated within 48 hours after extubation, they will be reassessed daily to identify the earliest time when they meet

study initial inclusion criteria once again so that screening for SBT readiness can resume. Patients will remain in the same group to which they were originally randomized. See Appendix 1.

**(iv) Criteria for Tracheostomy:** Patients who do not have a tracheostomy at study inclusion may receive a tracheostomy during their care, however, we will request in both groups that investigators, when possible, wait until at least day 10 before considering an elective tracheostomy [40,41]. To standardize care, we have developed criteria to guide performance of tracheostomy and weaning after a tracheostomy (Appendix 1).

**2.8 Challenges:** In this necessarily unblinded weaning trial, we will limit (i) *selection bias* by using central randomization with full allocation concealment, (ii) *identification bias* by having RTs lead screening and including an objective measure (f/$V_T$,) conducted on standardized settings in screening assessments, and (iii) *performance bias* by providing guidance statements on ventilator, PEEP and $FiO_2$ titration; NIV use, reintubation, and tracheostomy). To enhance generalizability, we will permit SBTs of 30 -120 min duration.

**2.9 Duration of Follow Up:** In all 4 groups, we will collect daily data up to successful extubation, ICU discharge, ICU death, or until day 60 after randomization (deemed ventilator dependent) whichever comes first. All patients will be followed to hospital discharge.

**2.10 Outcomes**

**2.10.1 Primary Outcome:** Aligned with our survey results [24] and informed by discussions with our PFAC, the primary outcome will be the *time to successful extubation* – signalling timely and safe liberation from the ventilator. Successful extubation is defined as the time when unsupported, spontaneous breathing BEGAN and was sustained for $\geq$ 48 hours after extubation/disconnection in patients with tracheostomy [38].

**2.10.2 Secondary Outcomes:** We prioritize secondary outcomes that are patient-centred [42] and important to citizens including: [1] Time to first passing an SBT, [2] ICU mortality, [3] Hospital and 90 day mortality [31], [4] Total duration of mechanical ventilation (invasive and noninvasive), [5] ICU length of stay, [6] Hospital length of stay, [7] Use of NIV after extubation, [8] VAP, [9] adverse events, self-extubation, tracheostomy, reintubation, prolonged ventilation at d14 and d21, ICU readmission [32,33], [10] proportion receiving sedation/ analgesia/ antipsychotics at key time points or [11]screened positive for delirium at key time points , [12] HRQoL (EuroQuol EQ-5D) 6 months after randomization [34], [13] functional status 6 months after randomization using the FIM [35-37]**.**

**2.11 Analytic Plan:** We will summarize baseline data using descriptive statistics [43]. All analyses will be performed adhering to the intention-to-treat principle.

**2.11.1 Primary Analysis:** Time-to event outcomes present special challenges because death is a competing risk and survivor only analyses are improper sub-groups. We will construct cumulative incidence curves to provide outcome estimates accounting for death for screening frequency and SBT technique. Cause-specific treatment effects will be depicted with hazard ratios (HR) (95% CI) from Cox models.

**2.11.2 Secondary Analysis:** We will report treatment effects in time to event analyses using HR and odds ratio (OR) with 95% CIs for binary outcomes and mean difference for continuous outcomes [43].

**2.11.3 Exploratory and Adjusted Analyses:** To assess the effect of age (continuous variable) by treatment interaction on the HR of time to successful extubation, we will construct a Cox regression model using a restricted cubic spline for age. Instead of arbitrarily assigning different levels for each age group, period, and cohort, a smoothing function or spline [collections of cubic polynomials joined smoothly at predefined number of points (knots)] will be created. The number of knots is expected to be between 3 and 5 but will be selected based on the sample size assuming the relationship with age will change gradually and smoothly. We will evaluate fitting using bootstrap techniques. As an extension of linear models, this technique allows for nonlinearities and interactions (between variables) that are more flexible than the linear contrasts traditionally used in regression models and is easier to depict and interpret [44]. In exploratory analyses, we will assess for an interaction between screening frequency and SBT technique. In adjusted analyses, we will consider variables (e.g., COPD, frailty score etc.) of potential prognostic importance.

**2.11.4 Interim Analyses:** Interim analyses for safety (adverse events) and efficacy (primary outcome) will be performed at 25%, 50% and 75% of accrual. Given the risks of stopping early for benefit, statistical significance will be declared using small p-values according to the O'Brien-Fleming boundaries for the primary outcome (time to successful extubation) and reintubation rate [45].

**2.11.5** In lieu of the emerging COVID-19 pandemic**,** we will enroll up to 250 additional critically ill patients who test positive for COVID-19 alongside the originally planned 760 (COVID-19 negative) patients. In this manner, we will be able to report on weaning and clinical outcomes in COVID-19 positive and COVID-19 negative patients combined and separately. The primary, secondary, exploratory, and adjusted analyses will report on 760 COVID-19 negative patients. We will also report the primary and secondary outcomes in COVID-19 positive patients alone and with both cohorts (COVID-19 positive and negative) combined.

**2.12 Sample Size Justification:** Most weaning trials report mean or median total duration of ventilation, weaning time, or ventilator free days which are confounded by pre-randomization duration of ventilator support, premature (unsuccessful) extubations, and weighting of deaths. Only 2 weaning trials, enrolling 144 and 92 patients invasively ventilated for ≥ 24 hours in mixed medical surgical ICUs, reported median time to successful extubation of 5 days [46] in the control groups, and 3 days [46] or 4 days [38] in the intervention groups. Similarly, a trial comparing two sedation minimization strategies in 430 patients invasively ventilated for > 48 hours reported median times to successful extubation of 7 days in both strategies [47]. These estimates align with median weaning times reported in the control [range: 4.0-5.0 days] and intervention groups [range: 1.6-4.0 days] of other sedation and weaning trials and our pilot trials [11,16,48,49]. Our sample size estimates are entirely contained within these estimates.

**2.12.1 Minimal Clinically Important Difference**: In our pilot trials, some patients in both arms were recognized to be breathing spontaneously on a morning screen, passed an SBT, and were extubated - saving a full day of ventilation. In addition, some patients randomized to 'at least twice daily' screening passed an SBT in the afternoon and were extubated at least 3/4 of a day earlier. Reducing the time to successful extubation by even a part of a day has important implications for patients, hospitals, and our health care system. For patients, each day of invasive ventilation increases their exposure to harmful co-interventions (sedation), risk for developing VAP, discomfort, and distress. Since most ICUs operate at or near capacity with a fixed number of beds, saving a partial or full day of invasive ventilation would enable care to be provided to other patients and transfers from other care settings (Emergency Departments, recovery units, hospital wards, and referring hospitals) reducing wait times, adverse events (e.g., length of stay) and providing a more equitable and ethical distribution of limited ICU resources [50-52]. While underpowered, our pilot trial estimates suggest that at least a full day of ventilation may be saved.

**2.12.2 Sample Size Estimate:** To take into consideration deaths before successful extubation, we used cumulative incidence curves generated from our pilot trials and computed 3 mortality HRs per patient day [HR 2.9% (Release Trial) and HR 3.3% (SENIOR Trial) and HR 3.2% (combined)] to compute the sample size [27]. To demonstrate a reduction in time to successful extubation from a median of 5.0 days to 4.0 days [HR 1.25] [38,46] with 80% power and α =0.05, and allowing for 3 interim analyses, the proposed trial would require 760 patients. *A priori,* we do not expect an interaction since mechanistically and sequentially an interaction is very unlikely. Since the groups are orthogonal, the main effects (in the absence of interactions) will have the same power to detect the same size differences (see Sample Size Table below).

| Median time (Once daily) | Median time (At Least Twice daily) | Hazard Ratio | Power | Alpha | N |
|---|---|---|---|---|---|
| 5.0 days | 4.2 days | HR 1.19 | 80% | 0.05 | 1244 |
| 5.0 days | 4.1 days | HR 1.22 | 80% | 0.05 | 960 |
| **5.0 days** | **4.0 days** | **HR 1.25** | **80%** | **0.05** | ***760** |
| 4.5 days | 3.4 days | HR 1.32 | 80% | 0.05 | 472 |
| 4.0 days | 3.5 days | HR 1.14 | 80% | 0.05 | 2064 |

**2.13 Timeline:** The trial will be conducted in 7 phases over 54 months: (i) start-up/recruitment (31 months), (ii) 6 month follow-up assessments (months 11 to 37), (iii) data cleaning (months 32-40), (iv) trial close out

(months 41-43), (v) trial analysis/manuscript preparation (months 44-48), (vi) follow-up outcomes close out (months 48-50), and (vii) follow-up analysis/manuscript preparation (months 51-54). We expect to recruit 2 patients per ICU per month. We allowed for variable REB approval times during start-up and for monthly recruitment to 'ramp up' (see Recruitment Table below). We plan to publish 3 manuscripts: (i) protocol and analytic plan (month 6), (ii) trial results (month 48), and (iii) HRQoL and functional status results (month 54).

| **Study Month** | **1** | **2** | **3** | **4** | **5** | **6** | **7** | **8** | **9** | **10** | **11** | **12** |
|---|---|---|---|---|---|---|---|---|---|---|---|---|
| Recruitment<br>No. ICUs | Start<br>up | Start<br>up | Start<br>up | Start<br>up | 2<br>[1] | 2<br>[1] | 4<br>[2] | 6<br>[6] | 10<br>[10] | 14<br>[14] | 18<br>[18] | 20<br>[20] |
| **Study Month** | **13** | **14** | **15** | **16** | **17** | **18** | **19** | **20** | **21** | **22** | **23** | **24** |
| Recruitment<br>No. ICUs | 24<br>[20] | 27<br>[20] | 30<br>[20] | 30<br>[20] | 30<br>[20] | 35<br>[20] | 35<br>[20] | 35<br>[20] | 40<br>[20] | 40<br>[20] | 40<br>[20] | 40<br>[20] |
| **Study Month** | **25** | **26** | **27** | **28** | **29** | **30** | **31** | **32** | **33** | **34** | **35** | **36-37** |
| Recruitment<br>No. ICUs | 40<br>[20] | 40<br>[20] | 40<br>[20] | 40<br>[20] | 40<br>[20] | 40<br>[20] | 38<br>[30] | Follow<br>up | Follow<br>up | Follow<br>up | Follow<br>up | Follow<br>up |

**3.0 Strengths of the Research Team:** Dr. Burns (Toronto, Canada) and Dr. Nicholas Hill (Boston, USA) are Co-PIs of the FAST Trial. Dr. Karen Burns leads a *Program of Research in Weaning* including many of the preparatory studies leading up to the FAST trial. While developing this research program, she held CIHR Clinician Scientist Awards (Phases 1 & 2) and an MRI career award. She has 75% of her time protected for research. Dr. Hill is an internationally recognized expert in NIV and weaning with an extensive track record of publications. He has held leadership positions in several critical care societies. Together, we assembled a team with expertise in mechanical ventilation and weaning (KB, DC, PD, AS, FL, NH, JD, MT, RB, RH, ML, GC), clinical trial design (KB, DC, AS, FL, NH, MT, RB, ML), and first hand ventilation experiences (PK, KK, MF, AG, SM). Members of our team have experience in leading multicenter studies (KB, DC, AS, FL, BR, NH, RB, JD, MT, ML, GC) and expertise in epidemiology (KT, KB, DC, AS, FL), quality and safety (PD, AA), knowledge translation (NH, PD, DC, RB), engagement (KB), and health policy (AG). Our team includes a RT (TP).

**3.1 Trial Organization, Research Environment and Resources**

**3.1.1 Trial Methods Centre**: The Applied Health Research Centre (AHRC; www.ahrconline.com ) of St. Michaels Hospital will serve as the data management and coordinating center. The AHRC is a not-for profit academic research organization affiliated with the University of Toronto. As a comprehensive trials unit it employs expert project staff with experience in RCT implementation and coordination. The Methods Centre will be responsible for developing and programming Medidata RAVE™, data monitoring, data management and analysis, and providing progress and data reports to the Steering Committee and DSMC. Professor Kevin Thorpe, head of biostatistics (AHRC) and a specialist in RCT analysis, will be our trial statistician.

**3.1.2 Steering Committee:** The Steering Committee will include the Co-Principal Investigators (Drs. Karen Burns and Nicholas Hill), 5 co-Investigators (Tom Piraino RRT, Dr. Peter Dodek, Dr. Andrew Seely, Dr. Maged Tanios, Dr. John Devlin), a PFAC member (Audrey Gouskos, MPA), and a research specialist from the AHRC. We will meet Q2 monthly by teleconference to discuss issues arising during trial implementation.

**3.1.3 Patient and Family Advisory Committee (PFAC):** As integral members of our team, the PFAC will serve in an advisory capacity to the Steering Committee. Our PFAC members include Audrey Gouskos (ICU survivor, Canada), Margaret Francis (family member, Canada) and Phyllis Kay (family member, USA), Susan Mitchell (SM), (family member, Canada), and Ken Kiedrowski (KK) (ICU survivor, USA) (see Support Letters). One PFAC member will be on the Steering Committee (AG) and another will serve on the DSMC (MF).

**3.1.4 Data Safety and Monitoring Committee (DSMC):** The DSMC will be chaired by Dr. Stefano Nava, a professor and senior researcher from Università di Bologna, Italy. Other DSMC members include Drs. Scott K. Epstein (intensivist, weaning expert, Associate Dean), Taylor Thompson (intensivist, mechanical ventilation expert), Dean Fergusson PhD (head, Clinical Epidemiology Methods Center, Ottawa Hospital Research Institute) and Margaret Francis (PFAC member).

**3.1.5 Knowledge Translation**: We have engaged key stakeholders to aid us with translating our findings into practice through conferences, websites, and using social media including research consortia (CCCTG, PETAL Network), respiratory care societies (CSRT, AARC), critical care societies (CCCS, ATS, SCCM), patient advisory groups (ATS, CCCTG), and government (Dr. B. Lawless, Critical Care Services Ontario).

**3.1.6 Participating ICUs and Personnel:** We will implement the FAST Trials in 10 ICUs in Canada and 10 ICUs in the USA (see Support Letters). The capacity to recruit patients from participating ICUs is supported by our pilot trial recruitment and a detailed evaluation of the number of beds and the average ICU admission rate per month. To enhance generalizability, we recruited both academic (n=17) and community (n=3) ICUs. All ICUs have experience in conducting mechanical ventilation research [27,53,54].

**4.0 Relevance:** The FAST Trial holds promise as a trial that will inform clinical decision-making and enhance care delivery in our ICUs, and improve the health outcomes of critically ill adults**.**

## References


1. Multz AS, Chalfin DG, Samson IM, et al. A “closed” medical intensive care unit improves resource utilization when compared to an “open” MICU. *Am J Respir Crit Care Med*. 1998;157:1468-73.

2. Needham D, Bronskill SE, Sibbald WJ. Pronovost PJ, Laupacis A. mechanical ventilation in Ontario, 1999-2000: incidence, survival and hospital bed utilization of noncardiac surgery adult patients. *Crit Care Med*. 2004;32:1504-1509.

3. Needham D, Bronskill SE, CalinawanJR, et al. Projected incidence of mechanical ventilation in Ontario to 2026: Preparing for the aging baby boomers. *Crit Care Med*. 2005;33:574-9.

4. Esteban A, Alia I, Ibanez J, Benito S and Tobin MJ. Modes of mechanical ventilation and weaning. A national survey of Spanish hospitals. The Spanish Lung Failure Collaborative Group. *Chest*. 1994;106:1188-93.

5. Pingleton SK. Complications of acute respiratory failure*. Am Rev Respir Dis.* 1988;137:1463-93.

6. Niederman MS, Ferranti RD, Ziegler A, Merrill W, Reynolds HY. Respiratory infection complicating long-term tracheostomy: the implication of persistent gram-negative tracheobronchial colonization. *Chest* 1984;85:39-44.

7. Heyland DK, Cook DJ, Griffith L, Keenan SP, Brun-Buisson C. The attributable morbidity and mortality of ventilator associated pneumonia in the critically ill patient. The Canadian Critical Care Trials Group. *Am J Respir Crit Care Med* 1999;159:1249-56.

8. MacIntyre NR, Cook DJ, Ely EW, Epstein SK, Fink JB, Heffner JE, et al. Evidence-based guidelines for weaning and discontinuing ventilatory support. A collective task force facilitated by the American College of Chest Physicians; the American Association for Respiratory Care; and the American College of Critical Care Medicine. *Chest* 2001;6 Suppl:375-95.

9. Ely EW, Baker AM, Dunagan DP, et al. Effect of the duration of mechanical ventilation on identifying patients capable of breathing spontaneously. *N Engl J Med.* 1996;335:1864-9.

10. Kollef MH, Shapiro SD, Silver P, et al. A randomized, controlled trial of protocol-directed versus physician-directed weaning from mechanical ventilation. *Crit Care Med.* 1997;25:567-74.

11. Marelich GP, Murin S, Battistella F, et al. Protocol weaning of mechanical ventilation in medical and surgical patients by respiratory care practitioners and nurses. Effect on weaning time and incidence of ventilator associated pneumonia. *Chest*. 2000;118:459-67.

12. Esteban A, Alia I, Tobin MJ. Effect of spontaneous breathing trial duration on outcome of attempts to discontinue mechanical ventilation. *Am J Respir Crit Care Med.* 1999;159:512-8.

13. Esteban A, Alia I, Gordo F, et al. Extubation outcome after spontaneous breathing trials with t-tube or pressure support ventilation. *Am J Respir Crit Care Med.* 1997;156:459-65.

14. Perren A, Domenighetti G, Mauri S, et al. Protocol-directed weaning from mechanical ventilation; clinical outcome in patients randomized for a 30-minute and 120-minute trial with pressure support. *Intensive Care Med*. 2002;28:1058-63.

15. Brochard L, Rauss A, Benito S, et al. Comparison of three methods of gradual withdrawal from ventilatory support during weaning from mechanical ventilation. *Am J Respir Crit Care Med.* 1994;150:896-903.

16. Esteban A, Frutos F, Tobin MJ, et al. A comparison of four methods of weaning patients from mechanical ventilation. *N Engl J Med*. 1995;332:345-50.

17. Esen F, Denkel T, Telci L, et al. Comparison of pressure support ventilation and intermittent mandatory ventilation during weaning in patients with acute respiratory failure. *Adv Exp Med Biol.* 1992;317:371-6.

18. Wood G, MacLeod B, Moffatt S. Weaning from mechanical ventilation: physician-directed vs. a respiratory-therapist-directed protocol. *Respir Care* 1995;40:219-24.

19 Torres A, Gatell JM, Aznar E, El-Ebiary M, Puig de la Bellacasa J, Gonzalez J, et al. Re-intubation increases the risk of nosocomial pneumonia in patients needing mechanical ventilation. *Am J Respir Crit Care Med* 1995; 152:137–41.

20. Blackwood B, Burns K, Cardwell C, O'Halloran P. Protocolized vs. non-protocolized weaning for reducing the duration of mechanical ventilation in critically ill adult patients Cochrane systematic review and meta-analysis. *Cochrane Database of Syst Reviews.* 2014; Issue 11: Nov 6;11:CD006904.

21. Boles JM, Bion J, Connors A, Herridge M, Marsh B, Melot C, Pearl R, et al. Task Force: Weaning From Mechanical Ventilation. *Eur Respir J* 2007; 29: 1033–1056.

22. Ladiera MT, Vital FM, Andriolo RB, Andriolo BN, Attalah AN, Peccin MS. Pressure Support vs. T-tube for weaning from mechanical ventilation. *Cochrane Database Syst Rev.* 2014; Issue 5:CD006056.

23. Burns KEA, Soliman I, Adhikari NKJ, Zwein A, Wong JTY, Gomez-Builes C, Pellegrini JA, Chen L, Rittayamai N, Sklar M, Brochard LJ, Friedrich JO. Is There a Preferred Method to Conduct a Spontaneous Breathing Trial? A Systematic Review and Meta-Analysis of Alternative Techniques. (Submitted: *Am J Respir Crit Care Med*).

24. Burns KE, Rizvi L, Jakob K. Sanu, Aguirre V, Gomes J, Mehta S. Citizen's Preferences Regarding Important Outcomes and Treatment Options While Removing Patients (Weaning) From Breathing Machines. (In Press: *Annals of the American Thoracic Society*).

25. Burns KEA, Lellouche F, Loisel F, Slutsky AS, Meret A, Smith O, Saskin R, Meade MO. Weaning Critically Ill Adults from Invasive mechanical Ventilation: A National Survey. *Can J Anaesth.* 2009 56(8):567-76.

26. Burns KEA, Raptis S, Bakshi J, Cook DJ, Jones A, Epstein S, Kapadia F, Wu YY, Gattas D, Cooper J, Lellouche F, Mekontso-Dessap A, Vaidyanathan P, Brochard L, Meret A, Slutsky A, Meade MO. Practice Variation in Weaning Critically Ill Adults from Invasive Mechanical Ventilation: Preliminary Results of an International Survey. *Am J Respir Crit Care Med* 183;2011:A6247.

27. Burns KEA, Wong J, Rizvi L, Hand L, Cook DJ, Dodek P, Mehta S, Kho ME, Friedrich JO, Seely AJ, Brochard L, Meade MO. Parallel Pilot Trials of Screening Frequency for Liberation from Mechanical Ventilation: The RELEASE and SENIOR Trial Protocols. *J Clin Trials* 2015 5(4).

28. Tri-Council Policy Working Party on Ethics. Code of ethical conduct for research involving humans. Ottawa. The Medical Research Council of Canada, the Natural Sciences and Engineering Research Council of Canada and the Social Sciences and Humanities Research Council of Canada, 1997. Available at www.ncehr-cnehr.org/english/code_2 (last accessed: Feb 11th, 2016).

29. Peigne V, Chaize M, Falissard B, et al. Important Questions Asked by Family Members of ICU Patients. *Crit Care Med*. 2011;39:1365-71.

30. Reay H, Arulkumaran N, Brett SJ. James Lind Alliance ICU Priority Setting Partnership Priorities for future Intensive Care Research in the UK; Results of a James Lind Alliance Priority Setting Partnership. *JICS* 2014;15:299-96.

31. Nisula A, Kaukonen KM, Vaara ST, Korhonen AM, Poukkanen M, Karlsson S, Haapio M, Inkinen O, Parviainen I. Incidence, risk factors and 90-day mortality of patients with acute kidney injury in Finnish intensive care units: the FINNAKI study. *Intensive Care Med*. 2013;39(3):420-8.

32.Frost SA, Alexandrou E, Bogdanovski T, Salamonson Y, Davidson PM, Parr MJ, Hillman KM Severity of illness and risk of readmission to intensive care: a meta-analysis. Resuscitation. 2009 May;80(5):505-10. doi: 10.1016/j.*Resuscitation*.2009.02.015. Epub 2009 Apr 1.

33. Rosenberg AL, Watts C. Patients readmitted to ICUs: a systematic review of risk factors and outcomes. *Chest.* 2000 Aug;118(2):492-502.

34. Herdman M, Gudex C, Lloyd A, et al. Development and preliminary testing of the new five-level version of EQ-5D (EQ-5D-5L). *Qual Life Res.* 2011;20:1727-36.

35. Weiss, D.S. (2007). The Impact of Event Scale: Revised. In J.P. Wilson, & C.S. Tang (Eds.), Cross-cultural assessment of psychological trauma and PTSD (pp. 219-238). New York: Springer. Polisher Research Institute. Instrumental Activities of Daily Living Scale (IADL). Available at: http://www.abramsoncenter.org/PRI/documents/IADL.pdf. (Last accessed October 19, 2015).

36. Oczkowski WJ, Barreca S. The functional independence measure: its use to identify rehabilitation needs in stroke survivors. *Arch Phys Med Rehabil* 1993; 74: 1291-4.

37. Koyama T, Matsumoto K, Okuno T, Domen K. Relationships between independence level of single motor-FIM items and FIM-motor scores in patients with hemiplegia after stroke: an ordinal logistic modelling study. *J Rehabil Med* 2006; 38: 280-6.

38. Burns KE, Meade MO, Lessard MR, Hand L, Zhou Q, Keenan SP, Lellouche F. Wean Earlier and Automatically with New Technology (The WEAN Study): A Multicentre Pilot RCT. *Am J Respir Crit Care Med.* 2013 Jun 1;187(11):1203-11.

39. Burns KEA, Premji A, Meade MO, Adhikari NKJ. Weaning Critically Ill Adults with Noninvasive Positive Pressure Ventilation: An Updated Systematic Review and Meta-Analysis. Review. *CMAJ.* 2014 Feb 18;186(3):E112-22.

40.Terragni PP, Antonelli M, Fumagalli R, Faggiano C, Berardino M, Pallavicini FB, et al. Early vs. Late Tracheotomy for Prevention of Pneumonia in Mechanically Ventilated Adult ICU Patients: A Randomized Controlled Trial. *JAMA.* 2010;303(15):1483-1489.

41. Young D, Harrison DA, Cuthbertson BH, Rowan K, for the Trachman Collaborators. Effect of Early vs. Late Tracheostomy Placement on Survival in Patients Receiving Mechanical Ventilation: The TracMan Randomized Trial. *JAMA.* 2013;309(20):2121-2129.

42. Curtis JR. The patient-centered' outcomes of critical care: what are they and how should they be used. *New Horiz.* 1998;6(1):26-32.

43. Streiner DL, Norman GR (2008). Health Measurement Scales - A Practical Guide to Their Development and Use. Oxford University Press, Oxford, UK, 4th edition.

44. Fan J & Yao, Q (2005). "Spline Methods". Nonlinear time series: nonparametric and parametric methods. Springer (New York, New York). p. 247.

45. O'Brien, P.C.; Fleming, T.R. (1979). "A Multiple Testing Procedure for Clinical Trials". *Biometrics* **35**: 549–556.

46. Lellouche F, Mancebo J, Jolliet P, Roeseler J, Schortgen F, Dojat M, Cabello B, Bouadma L, Rodriguez P, Maggiore S, Reynaert M, mersmann S, Brochard L. A Multicenter Randomized Trial of Computer-driven Protocolized Weaning from Mechanical Ventilation. Am J Respir *Crit Care Med*.2006; 174: 894–900.

47. Mehta S, Burry L, Cook D, Fergusson D, Steinberg M, Granton J, Herridge M, Ferguson N, Devlin J, Tanios M, Dodek P, Fowler R, Burns K, Jacka M, Olafson K, Skrobik Y, Hébert P, Sabri E, Meade M; SLEAP Investigators; Canadian Critical Care Trials Group. Daily sedation interruption in mechanically ventilated critically ill patients cared for with a sedation protocol: a randomized controlled trial. *JAMA*. 2012;308(19):1985-92. Erratum in: JAMA. 2013;309(3):237.

48. Bosma KJ, Read BA, Bahrgard Nikoo MJ, Jones PM, Priestap FA, Lewis JF. A Pilot Randomized Trial Comparing Weaning From Mechanical Ventilation on Pressure Support Versus Proportional Assist Ventilation. *Crit Care Med*. 2016;44(6):1098-108.

49. Brook AD, Ahrens TS, Schaiff R, Prentice D, Sherman G, Shannon W, Kollef MH. Effect of a nursing-implemented sedation protocol on the duration of mechanical ventilation. *Crit Care Med*. 1999 Dec;27(12):2609-15.

50. Huynh TN, Kleerup EC, Raj PP, Wenger NS. The opportunity cost of futile treatment in the ICU. *Crit Care Med*. 2014;42(9):1977-82.

51. Chalfin DB, Trzeciak S, Likourezos A, et al.DELAY-ED study group. Impact of delayed transfer of critically ill patients from the emergency department to the intensive care unit. *Crit Care Med*. 2007;35:1477-83.

52. Stelfox HT, Hemmelgarn BR, Bagshaw SM, Gao S, Doig CJ, Nijssen-Jordan C, Manns B. Intensive care unit bed availability and outcomes for hospitalized patients with sudden clinical deterioration. *Arch Intern Med*. 2012;172(6):467-74.

53. MacIntyre NR, Cook DJ, Ely EW, Epstein SK, Fink JB, Heffner JE, et al. Evidence-based guidelines for weaning and discontinuing ventilatory support. A collective task force facilitated by the American College of Chest Physicians; the American Association for Respiratory Care; and the American College of Critical Care Medicine. *Chest*. 2001;6 Suppl:375-95.

54. Kuhlen R, Max M, Dembinski R, Terbeck S, Jürgens E, Rossaint R. Breathing pattern and workload during automatic tube compensation, pressure support and T-piece trials in weaning patients. *Eur J Anaesthesiol.*2003;20(1):10-6.
