# Supplementary material for: Frequency of screening and SBT Technique Trial—North American Weaning Collaboration (FAST-NAWC): an update to the protocol and statistical analysis plan
Source: Trials. 2023 Oct 2;24:626. doi: 10.1186/s13063-023-07079-5 (PMC10544476; doi:10.1186/s13063-023-07079-5)
Supplement: Supplementary file 2 — Additional file 2: Appendix 2. SAP checklist in accordance with the Guidelines for the Content of Statistical Analysis Plans in Clinical Trials. [file 13063_2023_7079_MOESM2_ESM.pdf]

STATISICAL ANALYSIS PLAN for the FAST-NAWC Trial

Version: 1.2

Date: 04/11/2022

Protocol Title: **Frequency of Screening and SBT Technique Trial - North American Weaning Collaboration (FAST-NAWC): a protocol for a multicenter, factorial randomized trial**

Short title: FAST-NAWC Trial

Protocol date: 22/06/2020

Protocol version: 6.0

Trial Registration: Prospectively registered (21/11/2016) on Clinical Trials.gov (NCT02399267).

Karen E. A. Burns MD, FRCPC, MSc

Associate Professor, Clinician Scientist

Unity Health Toronto (St. Michael's Hospital)

4-045 Donnelly Wing

Toronto, Ontario, CANADA M5B 1W8

Document History

Version	Reason for Change	Date
Version 1.1	Clarification of statistical tests that will be used in all analyses.	28/07/2022
Version 1.2	Clarification of statistical approach to analyzing data for COVID-19 negative and positive patients.	04/11/2022

Signatures

Co-Chief Principal Investigator and Chair of Trial Steering Committee

Name, Affiliation	Signature	Date
Dr. Karen E. A. Burns<br><br>Unity Health Toronto – St. Michael's Hospital, Toronto, Canada		November 17, 2022

Co-Chief Principal Investigator

Name, Affiliation	Signature	Date
Dr. Nicholas Hill<br><br>Tufts Medical Center, Boston, USA		11-17-22

Trial Statistician, Statistical Analysis Plan Author

Name, Affiliation	Signature	Date
Dr. Myriam Lafreniere-Roula<br><br>Applied Health Research Centre<br>Li Ka Shing Knowledge Institute – St. Michael's Hospital, Toronto, Canada		November 17, 2022

Senior Statistician

Name, Affiliation	Signature	Date
Prof. Kevin E. Thorpe<br><br>Dalla Lana School of Public Health, University of Toronto, Toronto, Canada		November 17, 2022

This *Statistical Analysis Plan is* the property of the Dr. Karen E. A. Burns of Unity Health Toronto - St. Michael's Hospital in Toronto, Canada. This trial is being implemented under the auspices of the Canadian Critical Care Trials Group. No section of this document may be copied, published, or used for any purpose without appropriate acknowledgement and the prior written permission of Dr. Karen E. A. Burns.

This Statistical Analysis Plan (SAP) describes the planned analyses and reporting for The FAST-NAWC trial: A multicenter, factorial design randomized controlled trial with concealed allocation, will comparing the effect of both screening frequency (once vs. at least twice daily) and SBT technique [Pressure Support (PS) + Positive End-Expiratory Pressure (PEEP) vs. T-piece] on the time to successful extubation (primary outcome) in 760 critically ill adults who are invasively ventilated for at least 24 hours in 20 North American intensive care units.

The structure and content of this SAP provide sufficient detail to meet the requirements identified by the International Council for Harmonisation of Technical Requirements for Registration of Pharmaceuticals for Human Use (ICH): Guidance on Statistical Principles in Clinical Trials (1). All work planned and reported for this SAP will follow national and international guidelines for statistical practice (1, 2).

The planned analyses identified in this SAP will be included in future manuscripts. Additional exploratory analyses, not necessarily identified in this SAP, may be performed to support planned analyses. Any post-hoc or unplanned analyses not specified in this SAP will be clearly identified as such in the Final Statistical Report (FSR) and manuscripts for publication.

This SAP was written and reviewed by Dr. Karen Burns and the trial statisticians (Dr. Myriam Lafreniere-Roula, Mr. Kevin E. Thorpe), the FAST-NAWC Trial Steering Committee and clinical investigators from the FAST-NAWC Trial. All contributors were blinded to treatment allocations and treatment-related trial results and will remain so until the central database is locked and the final data are extracted for analysis. This document is submitted prior to the completion of study follow-up and the commencement of data analysis.

The following documents were reviewed when preparing this SAP:

1. Published Clinical Research Protocol for the FAST pilot trial & FAST-NAWC Trial (version 6.0) (3,4)
2. Electronic case report forms and the Data Management Plan for the FAST-NAWC trial.
3. The Operations Manual (Version 4.0) for the FAST-NAWC trial.
4. Safety Monitoring Committee (SMC) Charter for the FAST-NAWC trial
5. ICH Harmonised Tripartite Guideline on Statistical Principles for Clinical Trials (1).
6. ICH E9 (R1) Addendum on estimands and sensitivity analysis in clinical trials to the guideline on statistical principles for clinical trials (5).
7. ICH Harmonised Tripartite Guideline on Structure and Content of Clinical Study Reports (6).

Readers of this SAP are encouraged to read the Clinical Research Protocols for further details on trial design and implementation.

Abbreviations:

AC = assist control
ALTD = at least twice daily
APRV = airway pressure regulated volume
BMI = body mass index
CI = confidence interval
CONSORT = consolidated standards of reporting
COVID-19 = coronavirus disease of 2019
CPAP = continuous positive airway pressure
CV = coefficient of variation
FAST-NAWC = Frequency of Screening and SBT Technique Trial – North American Weaning Collaborative
FIM =Functional Independence Measure
FiO2 = fractional concentration of inspired oxygen
GCS = Glasgow Coma Scale
HR = Hazard ratio
HRQoL = Heath related quality of life
ICH = international council for harmonization
ICU = intensive care unit
ITT = intention-to-treat
MV = mechanical ventilation
NIV = noninvasive ventilation
OD = once daily
PaO2/FiO2 = partial pressure of arterial oxygen/ fractional concentration of inspired oxygen
PAV = proportional assist ventilation
PEEP = positive end-expiratory pressure
PRVC pressure regulated volume control
PS = pressure support
RCT = randomized controlled trial
REDCaP = research electronic data capture
RT = respiratory therapist
SAP = statistical analysis plan
SAS = statistical analysis systems
SBT = spontaneous breathing trial
SD = standard deviation
SIMV = synchronized intermittent mandatory ventilation
SpO2 = pulse oximetry oxygen saturation
USA = United States of America
VAP = ventilator associated pneumonia
VS = volume support

1.0 Study Design and Research Questions

1.1. Background

Weaning from invasive mechanical ventilation (MV) is the process during which the work of breathing is transferred from the ventilator back to the patient. Nearly 40% of the time spent on invasive MV is dedicated to weaning [7, 8]. Although invasive MV is effective in managing respiratory failure, its use is associated with the development of numerous complications including ventilator-associated pneumonia (VAP) and respiratory muscle weakness [9]. The risk for VAP increases after the fifth day of invasive MV, is associated with substantial morbidity, and may increase mortality [10]. Conversely, premature or failed attempts at extubation necessitating reintubation are also associated with greater risk of VAP **[11],** prolonged intensive care unit (ICU) stay, and increased mortality [12,13]. Consequently, in their efforts to minimize patient's exposure to invasive MV, clinicians are challenged by a "tradeoff" between the complications associated with protracted ventilation and the risks associated with failed attempts at extubation [14].

More than two decades of research support the use of specific strategies to limit invasive MV including: (1) the use of multidisciplinary screening protocols to identify appropriate candidates for a spontaneous breathing trial (SBT) [15, 16]; (2) the conduct of SBTs [12,13,17,18] in patients who pass screening criteria; and (3) the use of specific modes and techniques (reductions in Pressure Support [PS]) and once daily (OD) SBTs (PS ± positive end expiratory pressure [PEEP] or T-piece) [18–20] to discontinue ventilator support in patients who fail an initial SBT. In a 2014 Cochrane review of 17 RCTs (n =2434), use of a screening protocol to identify SBT candidates compared to usual care was associated with significant reductions in weaning time, duration of MV, and intensive care unit (ICU) stay [15]. However, the strength of the conclusions that could be made from this meta-analysis were limited by heterogeneous populations, individual study risk of bias, and comparison of OD screening (intervention arm) to usual care (control arm) in most included trials. Importantly, no trial in this review compared more frequent screening to daily screening. Only one trial (n = 385) compared twice daily screening to usual care and noted a significantly shorter duration of MV and a trend toward a lower VAP with twice daily screening [21]. In national and international weaning surveys, daily SBT screening is the current standard of care [22,23]. Notwithstanding, daily screening may be poorly aligned with the continuous care provided in most ICUs because it is not patient centered and disregards the impact that treatment interventions, initiated after morning patient care rounds (e.g. reducing sedation), may have on SBT screening efforts later in the day.

The preferred technique to conduct an SBT remains unclear. Although PS SBTs are more commonly used in North America, significant inter-institutional variability exists in how SBTs are conducted [24]. Two meta-analyses of randomized controlled trials (RCTs) compared PS and T-piece weaning, including, but not limited to, the conduct of SBTs, found beneficial effects of PS weaning [25,26]. Similarly, a meta-analysis that directly compared alternative SBT techniques [17] and two guidance documents [18, 27] support use of PS SBTs. Conversely, a physiologic meta-analysis favored use of T-piece SBTs [28]. Although a large body of evidence regarding weaning and SBT conduct exists, it remains insufficient to guide care regarding how frequent SBT screening should occur and the SBT technique that should be used. The around-the-clock availability of respiratory therapists (RTs) in most North American ICUs presents a unique opportunity to identify the optimal SBT screening frequency and SBT technique. In the Frequency of Screening and SBT Technique – North American Weaning Collaborative (FAST-NAWC) Trial,

we will compare the effect of different screening frequencies (OD vs. at least twice daily [ALTD]) and SBT techniques (PS + PEEP vs. T-piece) in critically ill adults on time to successful extubation [29].

1.2 Summary of Design

The Frequency of Screening and SBT Technique Trial - North American Weaning Collaboration (FAST-NAWC) trial is a multicenter, factorial design randomized controlled trial with concealed allocation. The study population will include critically ill adults who are invasively ventilated for at least 24 hours in 20 North American ICUs.

1.3 Primary Research Question

The primary research question is to compare the effect of screening frequency and of spontaneous breathing trial (SBT) technique on the time to successful extubation in critically ill adults who are invasively ventilated for at least 24 hours. The two screening frequencies compared will be OD vs. ALTD. The two SBT techniques compared will be pressure support plus positive end expiratory pressure (PS + PEEP) vs. T-piece.

1.4 Secondary Research Questions

The secondary research questions will be to estimate the impact of the alternative screening and SBT techniques on other clinically important outcomes listed in the Secondary Outcomes section below.

2.0 Study Methods

2.1 Study population

We will include 760 critically ill adults aged ≥ 18 years (USA) or ≥ 16 years (Canada) or admitted to an adult ICU in approximately 20 ICUs in North America.

2.2 Eligibility

We will include critically ill adults who: (1) have received invasive mechanical ventilation for ≥ 24 h; (2) are capable of initiating spontaneous breaths or triggering the ventilator to give a breath on ventilator modes commonly used in the ICU; (3) require a fractional concentration of inspired oxygen (FiO_2) ≤ 70%; and (4) PEEP ≤12 cm H_2O.

We will exclude patients who meet one or more of the exclusion criteria listed below

1. Brain death or expected brain death
2. Patients who have evidence of myocardial ischemia in the 24-h period before enrollment, except if current trend in troponin is downward AND it has been ≥ 24 h since last troponin peak or the patient has undergone a revascularization procedure and attending physician has no concerns regarding ongoing ischemia
3. Patients who have received continuous invasive mechanical ventilation for ≥ 2 weeks
4. Patients who have a tracheostomy in situ at the time of screening
5. Patients who are receiving sedative infusions for seizures or alcohol withdrawal
6. Patients who require escalating doses of sedative agents

7. Patients who are receiving neuromuscular blockers or who have known quadriplegia, paraplegia, or four-limb weakness or paralysis preventing active mobilization (e.g., active range of motion, exercises in bed, sitting at edge of bed, transferring from bed to chair, standing, marching in place, ambulating)
8. Patients who are moribund (e.g. at imminent risk for death) or who have limitations of treatment (e.g., withdrawal of support, do not reintubate order, however, do not resuscitate orders will be permitted)
9. Patients who have profound neurologic deficits (e.g., after cardiac or respiratory arrest, large intracranial stroke or bleed) or Glasgow Coma Scale (GCS) ≤ 6
10. Patients who are using modes that automate SBT conduct
11. Patients who are current enrolled in a confounding study that includes a weaning protocol, or
12. Patients who were previously enrolled in this trial
13. Patients who have already undergone an SBT or are on T-piece, or continuous positive airway pressure (CPAP) alone (without PS), or PS ≤ 8 cm H_2O regardless of PEEP, or other “SBT equivalent” settings immediately before randomization
14. Patients who have already undergone extubation (planned, unplanned [e.g., self, accidental]) during the same ICU admission.

2.3 Enrollment

Research personnel [research coordinators and/or respiratory therapists (RTs)] will identify, consent, and enroll eligible patients from Monday to Friday during regular hours using a central randomization system, stratified by ICU with variable undisclosed block sizes. With the factorial design, patients will be randomized to both a screening frequency (OD vs. ALTD) and an SBT technique (PS + PEEP vs. T-piece).

2.4 Consent

This protocol was approved by the research ethics board of St. Michael’s Hospital (Toronto) and of participating ICUs. Given the minimal risk associated with the interventions being evaluated and the need to enroll patients as soon as possible after they can either initiate spontaneous breaths or trigger breaths, we will request ethics approval to use a hybrid consent model that prioritizes obtaining consent from patients (with decision-making capacity) or SDMs (when available) and permits deferred consent in their absence. For patients who are enrolled under deferred consent [30], research personnel will obtain consent as soon as possible after randomization. We have used this hybrid consent model in two multicenter, pilot, screening frequency trials comparing OD and ALTD screening [31].

3.0 Study Interventions

3. 1 Screening for readiness to undergo a spontaneous breathing trial

In the OD arm, RTs will screen study patients daily between 06:00 and 08:00 h. In the ALTD arm, patients will be screened at least twice daily between 06:00 and 08:00 h and between 13:00 and 15:00 h; additional screening will be permitted at the discretion of the ICU team. If a screening period is missed (inadvertently or due to an operation/ procedure necessitating absence from the ICU), it may be conducted later on the same day and ideally within 6 h of the scheduled screening period. For patients randomized after 10:00 h, only one screening assessment will be required on the first study day regardless of study arm.

To pass a screening assessment and undergo an SBT, all of the following criteria must be met:

1. The patient must be capable of initiating spontaneous breaths on PS or Proportional Assist Ventilation (PAV) or triggering breaths on volume or pressure Assist Control (AC), volume or pressure synchronized intermittent mandatory ventilation (SIMV) ± PS, pressure regulated volume control (PRVC), volume support (VS), or Airway pressure regulated volume (APRV);
2. The ratio of partial pressure of oxygen to FiO_2 (PaO_2/FiO_2) ≥ 200 mmHg;
3. Respiratory rate ≤ 35 breaths/min;
4. PEEP ≤ 10 cm H_2O;
5. Heart rate ≤ 140 beats/min;
6. The ratio of respiratory frequency to tidal volume (f/VT) < 105 breaths/min/L [32] during a 2 min assessment on Continuous positive airway pressure (CPAP) of 0 cm H_2O (alternatively PS = 0 cm H_2O /PEEP = 0 cm H_2O).

3.2 Conduct of spontaneous breathing trials

After passing a screening assessment, patients will undergo an initial SBT according to treatment assignment (PS + PEEP vs. T-piece). All SBTs will be 30–120min in duration with the actual duration selected by clinicians [13,27]. SBTs will be conducted on T-piece (off ventilator with no CPAP/ PEEP) or with PS > 0 and ≤ 8 cm H_2O with PEEP > 0 and ≤ 5 cm H_2O [27]. Between SBT trials, patients will be returned to the mode of ventilation used before the SBT, unless criteria are met to remain on/return to a mode of support that assumes no spontaneous or triggered breaths. We will use standardized criteria to determine SBT failure in both arms [33]. After an unsuccessful SBT, patients will be returned to the ventilator settings used before the SBT and ventilator settings will be adjusted to restore respiratory comfort.

3.3 Extubation

Patients who pass an SBT will be assessed for extubation. Extubation should be performed as soon as possible after passing an SBT. To be extubated patients should meet all criteria depicted below.
As this was not a trial focused on extubation, we did not protocolize extubation. Conversely, we will record the time that patients met criteria for extubation and the time patients were actually extubated.

Extubation Criteria
(1) SpO_2 ≥ 90% or at baseline level in chronically hypoxemic patients on an FiO_2 ≤ 40% and PEEP ≤ 5 cm H_2O
(2) A cough of sufficient strength to clear secretions and must not require suctioning more than every 2 hours
(3) Patients should be hemodynamically stable (off vasopressors or on minimal levophed, i.e. ≤ 7 μg/min [0.1 μg/kg/min or equivalent])
(4) A level of consciousness sufficient to ensure airway protection and
(5) A cuff leak is present
All of the above criteria (except nos. 4 and 5) will also apply to patients who undergo trach mask trials and are disconnected.

3.4 Other considerations

We provide criteria to suspend the protocol and return to a fully controlled mode of ventilation [protocol]. We standardized approaches to ventilator titration, use of noninvasive (NIV) after extubation, reintubation, and tracheostomy [34,35]

4.0 Outcomes

4.1 Primary Outcome

The primary outcome is the time to successful extubation.

4.2 Secondary Outcomes

The secondary outcomes are time to first passing an SBT, ICU mortality, hospital and 90-day mortality, total duration of mechanical ventilation (invasive and non-invasive), ICU length of stay Hospital length of stay use of NIV after extubation, VAP, adverse events including self-extubation, tracheostomy, reintubation, prolonged ventilation (patients remaining intubating at day 14 and/or day 21), ICU readmission, proportion of patients who receive sedation, analgesia, antipsychotics at key time points, proportion who screen positive for delirium at key time points. In addition, we will report health related quality of life (HRQol) using the EuroQuol EQ-5 and functional status using the Functional Independence Measure (FIM) at 6-months after randomization.

5.0 Sample size estimation

To compute sample size and take into consideration deaths that occur before successful extubation, we used cumulative incidence curves generated from our pilot trials and computed three mortality hazard ratios (HRs) per patient day (HR 2.9 [Release Trial] and HR 3.3 [SENIOR Trial] and HR 3.2 [combined]) [31]. We will require 760 patients to demonstrate a reduction in time to successful extubation from a median of 5.0 days to 4.0 days (HR 1.25) [33, 36] with 80% power and $\alpha = 0.05$ and allowing for three interim analyses. *A priori,* we do not expect an interaction since mechanistically and sequentially an interaction is unlikely. Since the groups are orthogonal, the main effects (in the absence of interactions) will have the same power to detect the same size differences.

6.0 Statistical Samples

6.1 Intention to Treat Sample

The intention-to-treat (ITT) sample will be comprised of all randomized patients, irrespective of compliance to their randomized group. Patients will contribute outcome data to the analysis based on the randomized group.

6.2 Per-Protocol Sample

We will conduct two per-protocol analyses. The first per-protocol sample will be comprised of patients who underwent all screens and SBTs using their assigned strategies. The second per-protocol analysis, will be comprised of patients who underwent 80% or more of their screens and SBTs using their assigned strategies.

6.3 Safety Sample

The safety sample will be comprised of all randomized patients.

7.0 Statistical Analysis and Considerations

7.1 Primary Outcome

The primary outcome is time to successful extubation. The cumulative incidence of successful extubation will be estimated accounting for the competing risk of death. The hazard ratio of successful extubation across groups will be estimated using a cause-specific hazard model. Treatment effects will be expressed as hazard ratios with 95% confidence intervals (Cis).

7.1 A Primary Analysis

The primary analysis will be an unadjusted analysis of time to successful extubation using cause-specific Cox regression to estimate the main effects of screening frequency and SBT technique.

7.2 Secondary Analyses

Secondary analyses will include an exploratory analysis of interaction between the main effects as well as adjusted analyses for factors of prognostic significance. In particular, the effect of age will be assessed using restricted cubic splines to allow for nonlinear effects and the effect of other factors of prognostic importance such as sex, body mass index (BMI), chronic obstructive pulmonary disease (COPD), congestive heart failure (CHF), postoperative status, and frailty will also be assessed.

7.2 A Secondary Outcomes

Time-to-event secondary outcomes will be analyzed using the same approach as that used for the primary outcome and treatment effects will be expressed as hazard ratio with 95% CIs.

Count variable outcomes (e.g. duration of ventilation, hospital length of stay) will be inspected to assess their distribution. They will be analyzed using Poisson regression if applicable or other more appropriate methods if they exhibit characteristics that warrant it (e.g., zero-inflation, overdispersion). Incidence rate ratios will be reported with their 95% CIs.

Continuous outcomes will be analyzed using generalized linear models and the treatment effects will be expressed as mean differences with their 95% confidence intervals. Categorical outcomes will be analyzed using generalized linear models with a logit link and odds ratio will be reported with their 95% Cis.

For all secondary outcomes, the primary analysis will be unadjusted and adjusted analyses may be performed as secondary analyses as appropriate.

7.3 Exploratory Analyses and Adjusted Analyses

To assess the effect of age (continuous variable) by treatment interaction on the HR of time to successful extubation, we will construct a Cox regression model using a restricted cubic spline for age. Instead of arbitrarily assigning different levels for each age group, period, and cohort, we will create a smoothing function or spline (collections of cubic polynomials joined smoothly at a predefined number of points [knots]). The number of knots is expected to be between three and five but will be selected based on the sample size assuming that the relationship with age will change gradually and smoothly. We will evaluate fit using bootstrap techniques. This technique allows for non-linearities and interactions between

variables that are more flexible than the linear contrasts traditionally used in regression models and is easier to depict and interpret [37]. In exploratory and adjusted analyses, we will assess for an interaction between screening frequency and SBT technique and variables [e.g. sex, BMI, COPD, CHF, postoperative status, frailty etc.) of potential prognostic importance.

A priori, we plan to conduct exploratory analyses to evaluate the impact of the alternative treatment strategies (OD vs. ALTD and PS + PEEP vs. T-piece) on time to first SBT (as opposed to first successful SBT) We will also examine the impact of the duration of invasive ventilation prior to randomization and first SBT on the outcomes of time to successful extubation (primary outcome) and time to first successful SBT (secondary outcome). To conduct these analyses, we will utilize the adjusted model (not including the variables of interest) to estimate the predicted value of the desired outcome(s). We will then examine the association between the fitted (predicted) value and the desired outcome (e.g., time to first SBT).

7.3 Handling of Incomplete Data

It is important to realize that the problem of incomplete data is distinct from adherence to the ITT principle. The ITT philosophy is to attribute the available patient data to the group they were randomized to, irrespective of compliance to randomization. However, it is not possible to analyze data that you do not have. Therefore, we will begin with the complete case analysis. However, this analysis can be biased, although if missing data is minimal, less than 5% say, bias will be minimal and conclusions will not be affected by more sophisticated approaches.

There is controversy regarding the appropriateness of imputing outcome data in clinical trials. Therefore, even if loss to follow-up (i.e. missing outcome) in the FAST-NAWC trial exceeds 5% no imputation methods will be employed for post-randomization outcomes. Note that for time-to-event outcomes, death prior to achieving the primary outcome will be treated as a competing risk. Subjects lost to follow-up for other reasons will be censored at the last known follow-up.

Multiple imputation may be employed to handle missing covariate data in secondary or adjusted analyses. We will report if imputation is used for covariates in secondary or adjusted analyses.

7.4 Inverse Probability Weighted Analysis

In the event there is enough missing outcome data to cause concern for the complete case analysis, an inverse probability weighted analysis will be conducted. This will be a two-step process.

First, a logistic regression model will be fitted to the complete ITT sample. The outcome for this model is 1 if the follow-up data (outcome) is present and 0 if it is missing. A list of baseline variables considered most likely to predict drop-out will be identified and used as covariates in the logistic regression model. The goal will be to create a model that is able to predict which patient was able (vs. unable) to complete the trial. Once the model is fitted, we will use it to generate predicted probabilities of XXXX "not being missing" for each patient.

The second stage proceeds on the complete case data as before, however the reciprocals of the predicted probabilities from the previous stage will be used as regression weights. The weighted analysis attempts to correct for the selection bias that could exist in the complete case analysis.

7.5 Regarding P-Values and Statistical Significance

Controversy exists regarding the use of p-values and significance testing. The issues are not particularly related to the p-values themselves but arise in the mechanical comparison to an arbitrary threshold (e.g., $p < 0.05$) which categorizes results into, “statistically significant” or “not statistically significant.” It is in this binary decision that Type 1 error may arise and the risk of making one or more Type 1 errors among all tests increases with the number of tests. It is this problem that leads many to adjust p-values (or equivalently, the threshold per test) in analysis.

However, the p-value as it stands simply represents a continuous measurement of the strength of the statistical evidence one has against some hypothesis (no difference between groups in this instance. It is thus best statistical practice to report for all outcomes analyzed, the estimated treatment effect, the 95% CI (or some other suitable level) and the p-value. This is the practice that will be adopted in the analyses. This gives a reader the required information to place the results into context. Note that the policy of some journals to request only the CI does not solve the problem it is intended to solve. Specifically, a reader will still mentally make the “significance conclusion” entirely using the CI.

8.0 Sequence of Planned Analyses

8.1 Interim Analyses

Three interim analyses were planned and performed at 25%, 50%, and 75% of accrual and statistical significance was declared using small p-values according to the O’Brien-Fleming boundaries for the primary outcome and for reintubation rate.

8.2 Final Analysis and Reporting

Planned analyses identified in the trial protocol and this SAP will be performed only after the last patient has completed the 6-month follow-up assessment visit, the REDCap™ database has been cleaned and locked, and results from separate analysis for protocol violations and deviations have been completed and declared final. Blinded data review meetings will be held before locking the REDCap ™ database and, again, before declaring final electronic copies of the data in Excel spreadsheets. There will be no un-blinded review and analyses will not commence until this SAP has been approved by the Lead Principal Investigators and Trial Statisticians and reviewed and approved by the trial Steering Committee.

Key statistics and trial results from the final analyses will be presented to the Steering Committee for discussion prior to completion of the final statistical report and subsequent manuscripts. Any post -hoc exploratory analyses performed to provide support for planned analyses but not identified in this SAP will be documented and reported in appendices and clearly identified as unplanned analyses. All analyses and their interpretation will be conducted independently of the trial funder - the Canadian Institutes of Health Research.

9.0 Impacts due to COVID-19

The COVID-19 (coronavirus disease of 2019) pandemic has impacted implementation of the FAST-NAWC trial. The study protocol was modified to address the study population (inclusion of covid-positive patients), conduct of SBTs in COVID-19 positive patients (aligned with commonly used infection control

practices), and analysis sections (to manage data from COVID-19 negative and positive patients). The details are presented in the accompanying update.

10.0 Protocol violations and protocol deviations

10.1 Protocol violations

We will assess and report major protocol violations reflecting compliance with the assigned screening frequency (OD vs. ALTD) and SBT technique (PS + PEEP vs. T-piece). We will report major protocol violations (related to screening frequency and SBT technique) by assigned treatment group.

Major protocol violations will include:

1. Screening period that was missed or an additional screening period that was conducted in the OD arm
2. Screening period that was missed in the ALTD screening arm.
3. Use of another SBT technique as opposed to the assigned SBT technique.

Minor protocol violations will include:

1. Patients that were NOT screened at the specified times (plus 6 hours – permitted by the protocol).
2. When an RSBI was not done or conducted on alternative ventilator settings.
3. Patients who underwent an SBT when they did NOT meet all SBT criteria.
4. Patients who underwent an SBT without a prior screening period.
5. Patients who did NOT undergo an SBT when all SBT criteria were met.

10.2 Protocol deviations

We recognize the need to encourage, monitor and document protocol adherence during implementation of RCTs. Reporting of protocol deviations is especially challenging, from time and resource perspectives, in trials evaluating interventions that require frequent (intermittent or continuous) monitoring or titration. (38) Conversely, inadequate reporting may prevent the identification of performance biases and influence the interpretation of the results, especially in unblinded trials. (38) In the FAST-NAWC trial, two individuals will adjudicate each screening opportunity and SBT conducted to assess for 'justifiable' reasons why a screen was not conducted as outlined in the protocol. We will maintain an Excel file (Microsoft, Redmond, Washington, USA) to record protocol violations and deviations. To ensure consistency in recording protocol deviations, we will maintain a log or permissible protocol deviations during the adjudication process.

11.0 Data manipulation and computing

All data manipulation, tables, figures, listings and analyses will be documented in SAS®, Stata® or R programs and performed using SAS version 9.4 or later, Stata version 17 or later, or R version 4.0.0 or later.

12.0 Reporting

We will summarize results from statistical analyses in tables, figures, and appendices as deemed appropriate by the members of the Steering Committee and the trial statisticians.

12.1 Trial profile

A CONSORT-style flow diagram will illustrate patient progression through the trial from initial screening for eligibility to completion of the final trial outcome. We will report separately on the 6-month follow-up assessments of HRQoL and FIM scores obtained through interviews of patients, SDMs, or legally authorized representatives who provide consent to participate unless required by the publishing journal of the parent trial.

We will report the number (percentage) of participants randomised to each treatment group along with reasons for study discontinuation (death, withdrawals, transfer to another (nonparticipating centre e.g., hospital or weaning facility), losses to follow-up, or other) by treatment group.

12.2 Patient characteristics and baseline comparisons

Demographic and other baseline characteristics will be summarised by assigned treatment group (OD vs. ALTD screening) and SBT technique (PS+PEEP vs. T-piece). Additionally, we will present results based on COVID-19 status (positive or negative) in either the main manuscript and/or the supplementary appendices.

We will summarize categorical variables by frequencies and percentages. Percentages will be calculated according to the number of patients for whom data are available. Where values are missing, the denominator, which will be less than the number of patients assigned to the treatment group, will be reported either in the body or a footnote in the summary table. Depending on their distribution, continuous variables will be summarised by mean and standard deviation (SD) as well as quartiles and interquartile ranges.

12.3 General reporting conventions

All tables and figures will be presented in portrait or landscape orientation depending on the easiest format to view. Legends will be used for all figures with more than one variable or item displayed. Figure lines will be wide enough to see the line after being copied.

All titles will be centred on individual pages. The first title line will be the number of the table, figure, or data listing. The second (and if required, third) line will be the description of the table, figure, or data listing. We will use the ICH numbering convention for all tables and figures (6).

12.4 Statistical summary conventions

For tables, sample sizes for each treatment group will be presented as totals in the column header (N=xxx), where appropriate. Sample sizes shown with summary statistics are the number (n) of patients with non-missing values.

Summaries for categorical variables will include only categories for which patients had a response in. All summaries for continuous variables will include: N, mean, SD, and quartiles. Other summaries (e.g., 95% confidence intervals, coefficient of variation (CV) or %CV will be used as appropriate (e.g., reporting hazard ratios or risk ratio). All percentages will be rounded and reported to a single decimal place (xx.x%). For percentages that are reported as integers, we will report percentages greater than 0% but <1% as <1%, whereas percentages greater than 99% but <100% will be reported as >99%. A percentage of 100% will be reported as 100%. Summaries that include p-values will report the p-value to three decimal places with a leading zero (0.001). P-values <0.001 will be reported as <0.001.

12.5 Trial Master File

The statistical master file will be held at the Applied Health Research Centre (St. Michael's Hospital, Toronto, Canada).

References

1. ICH. ICH Harmonised Tripartite Guideline: Statistical Principles for Clinical Trials E9. 1998

2. ASA. Ethical guidelines for statistical practice. Prepared by the Committee on Professional Ethics.1999.

3. Burns KEA, Rizvi L, Dodek P, Lamontagne F, Seely AJE, Rochwerg B, Tanios M, Piraino T, Honig E, Cirone R, Cook DJ. Frequency of screening and 'Spontaneous Breathing Trial' Technique Study (The FAST Trial): Design of a Multicentre, Pilot, Factorial Randomized Controlled Trial. 2016. Clin Trials 6: 284.

4. Burns KEA, Rizvi L, Cook DJ, Seely A, Rochwerg B, Lamontagne F, Devlin J, Dodek P, Mayette M, Tanios M, Gouskos A, Kay P, Mitchell S, Kiedrowski K, Hill N. Frequency of Screening and SBT Technique Trial – North American Weaning Collaborative: A Protocol for a Multicente, Factorial Randomized Trial. Trials 2019; 20(1):587. doi: 10.1186/s13063-019-3641-8.

5. ICH. ICH E9 (R1) Addendum on estimands and sensitivity analysis in clinical trials to the guideline on statistical principles for clinical trials. 2020.

6. ICH Harmonised Tripartite Guideline on Structure and Content of Clinical Study Reports E3. 1995.

7. Esteban A, Alia I, Ibanez J, Benito S, Tobin MJ. Modes of mechanical ventilation and weaning. A national survey of Spanish hospitals. The Spanish Lung Failure Collaborative Group. Chest. 1994;106:1188–93.

8. Esteban A, Anzueto A, Frutos F, et al. Characteristics and outcomes in adult patients receiving mechanical ventilation: a 28-day international study. JAMA. 2002;287(3):345–55.

9. Pingleton SK. Complications of acute respiratory failure. Am Rev Respir Dis. 1988;137:1463–93.

10. Heyland DK, Cook DJ, Griffith L, Keenan SP, Brun-Buisson C. The attributable morbidity and mortality of ventilator associated pneumonia in the critically ill patient. The Canadian Critical Care Trials Group. Am J Respir Crit Care Med. 1999;159:1249–56.

11. Thille AW, Harrois A, Schortgen F, Brun-Buisson C, Brochard L. Outcomes of extubation failure in medical intensive care unit patients. Crit Care Med. 2011;39:2612–8.

12. Esteban A, Alia I, Gordo F, et al. Extubation outcome after spontaneous breathing trials with t-tube or pressure support ventilation. Am J Respir Crit Care Med. 1997;156:459–65.

13. Esteban A, Alia I, Tobin MJ. Effect of spontaneous breathing trial duration on outcome of attempts to discontinue mechanical ventilation. Am J Respir Crit Care Med. 1999;159:512–8.

14. MacIntyre NR, Cook DJ, Ely EW Jr, Epstein SK, Fink JB, Heffner JE, et al. Evidence based guidelines for weaning and discontinuing ventilator support. A collective task force facilitated by the American College of Chest Physicians, the American Association for Respiratory Care and the College of Critical Care Medicine. Chest. 2001;120(Supple 6):375S–95S.

15. Blackwood B, Burns K, Cardwell C, Lavery G, O'Halloran P. Use of weaning protocols for reducing duration of mechanical ventilation in critically ill Burns et al. Trials (2019) 20:587 Page 7 of 8 adult patients: Un updated Cochrane systematic review and meta-analysis. Cochrane Database Syst Rev. 2014;Issue 11:CD006904. https://doi.org/10.1002/14651858.CD006904.

16. Perren A, Domenighetti G, Mauri S, et al. Protocol-directed weaning from mechanical ventilation; clinical outcome in patients randomized for a 30-minute and 120-minute trial with pressure support. Intensive Care Med. 2002;28:1058–63.

17. Burns KE, Soliman I, Adhikari NKJ, Zwein A, Wong JTY, Gomez-Builes C, et al. Trials directly comparing alternative spontaneous breathing trial techniques: a systematic review and meta-analysis. Crit Care. 2017;21(1):127.

18. Girard TD, Alhazzani W, Kress JP, Ouellette DR, Schmidt GA, Truwit JD, et al. An Official American Thoracic Society/American College of Chest Physicians Clinical Practice Guideline: Liberation from Mechanical Ventilation in Critically Ill Adults Rehabilitation Protocols, Ventilator Liberation Protocols, and Cuff Leak Tests. Am J Respir Crit Care Med. 2017;195(1):120–33.

19. Brochard L, Rauss A, Benito S, et al. Comparison of three methods of gradual withdrawal from ventilatory support during weaning from mechanical ventilation. Am J Respir Crit Care Med. 1994;150:896–903.

20. Esteban A, Frutos F, Tobin MJ, et al. A comparison of four methods of weaning patients from mechanical ventilation. N Engl J Med. 1995; 332:345–50.

21. Marelich GP, Murin S, Battistella F, et al. Protocol weaning of mechanical ventilation in medical and surgical patients by respiratory care practitioners and nurses. Effect on weaning time and incidence of ventilator associated pneumonia. Chest. 2000;118:459–67.

22. Burns KEA, Lellouche F, Loisel F, Slutsky AS, Meret A, Smith O, et al. Weaning critically ill adults from invasive mechanical ventilation: a national survey. Can J Anaesth. 2009;56(8):567–76.

23. Burns KE, Raptis SR, Nisenbaum R, Rizvi L, Jones A, Bashik J, et al. International practice variation in weaning critically ill adults from invasive mechanical ventilation. Ann Am Thorac Soc. 2018;15(4):494–502. https://doi. org/10.1513/AnnalsATS.201705-410OC.

24. Godard S, Herry C, Westergaard P, Scales N, Brown SM, Burns K, et al. Practice variation in spontaneous breathing trial performance and reporting. Can Respir J. 2016;2016:9848942. https://doi.org/10.1155/2016/9848942.

25. Ladiera MT, Vital FM, Andriolo RB, Andriolo BN, Attalah AN, Peccin MS. Pressure Support vs. T-tube for weaning from mechanical ventilation. Cochrane Database Syst Rev. 2014;Issue 5:CD006056.

26. Pellegrini JA, Moraes RB, Maccari JG, de Oliveira RP, Savi A, Ribeiro RA, et al. Spontaneous breathing trials with T-piece or pressure support ventilation. Respir Care. 2016;61(12):1693–703.

27. Boles JM, Bion J, Connors A, Herridge M, Marsh B, Melot C, Pearl R, et al. Task Force: weaning from mechanical ventilation. Eur Respir J. 2007;29:1033–56.

28. Sklar MC, Burns K, Rittayamai N, Lanys A, Rauseo M, Chen L, et al. Effort to breathe with various spontaneous breathing trial techniques. A physiological meta-analysis. Am J Respir Crit Care Med. 2017;195(11):1477–85. https://doi.org/10.1164/rccm.201607-1338OC.

29. Burns KE, Jacob SK, Aguirre V, Gomes J, Mehta S, Rizvi L. Stakeholder engagement in trial design: survey of visitors to critically ill patients regarding preferences for outcomes and treatment options during weaning from mechanical ventilation. Ann Am Thorac Soc. 2016;13(11):1962–8.

30. Tri-Council Policy Working Party on Ethics. Code of ethical conduct for research involving humans. Ottawa. The Medical Research Council of Canada, the Natural Sciences and Engineering Research Council of Canada and the Social Sciences and Humanities Research Council of Canada, 1997. www.ncehr-cnehr.org/english/code_2. Accessed 11 Feb 2016).

31. Burns KEA, Wong JTY, Dodek P, Cook DJ, Lamontagne F, Cohen A, et al. Frequency of screening for weaning from mechanical ventilation: two contemporaneous proof-of-principle randomized controlled trials. Crit Care Med. 2019;47:817–25. https://doi.org/10.1097/CCM.0000000000003722.

32. Yang KL, Tobin MJ. A prospective study of indexes predicting the outcome of trials of weaning from mechanical ventilation. N Engl J Med. 1991;324:1445–50.

33. Burns KE, Meade MO, Lessard MR, Hand L, Zhou Q, Keenan SP, et al. Wean Earlier and Automatically with New Technology (The WEAN Study): a multicentre pilot RCT. Am J Respir Crit Care Med. 2013;187(11):1203–11.

34. Terragni PP, Antonelli M, Fumagalli R, Faggiano C, Berardino M, Pallavicini FB, et al. Early vs. late tracheotomy for prevention of pneumonia in mechanically ventilated adult ICU patients: a randomized controlled trial. JAMA. 2010;303(15):1483–9.

35. Young D, Harrison DA, Cuthbertson BH, Rowan K, for the Trachman Collaborators. Effect of early vs. late tracheostomy placement on survival in patients receiving mechanical ventilation: the TracMan randomized trial. JAMA. 2013;309(20):2121–9.

36. Lellouche F, Mancebo J, Jolliet P, Roeseler J, Schortgen F, Dojat M, et al. A multicenter randomized trial of computer-driven protocolized weaning from mechanical ventilation. Am J Respir Crit Care Med. 2006;174:894–900.

37. Fan J, Yao Q. “Spline Methods”. Nonlinear time series: nonparametric and parametric methods. New York: Springer; 2005. p. 247.

38. Lauzier F, Adhikari NA, Seely A, Koo K, Belley-Cote E, Burns KEA, Cook DJ, D’Aragaon F, Rochwerg B, Kho M, Oczkowski SJW, Duan E, Meade MO, Day AG, Lamontagne F for the Academy of Critical Care – Development, Evaluation and Methodology. Protocol Adherence for Continuously Titrated Interventions in Randomized Trials: Reflections from the OVATION Pilot Trial. BMC Med Res Methodol. 2017 Jul 17;17(1):106. doi: 10.1186/s12874-017-0388-3.
